# The K^+^ channel GIRK2 is both necessary and sufficient for peripheral opioid-mediated analgesia

**DOI:** 10.1002/emmm.201201980

**Published:** 2013-07-01

**Authors:** Dinah Nockemann, Morgane Rouault, Dominika Labuz, Philip Hublitz, Kate McKnelly, Fernanda C Reis, Christoph Stein, Paul A Heppenstall

**Affiliations:** 1 Klinik für Anaesthesiologie und Operative Intensivmedizin, Freie Universität Berlin, Charité Campus Benjamin FranklinHindenburgdamm 30, Berlin, Germany; 2 Mouse Biology Unit, European Molecular Biology Laboratory (EMBL)Via Ramarini 32, Monterotondo, Italy

**Keywords:** Dorsal root ganglia, Ion channel, Opioids, Pain, Peripheral analgesia

## Abstract

The use of opioid agonists acting outside the central nervous system (CNS) is a promising therapeutic strategy for pain control that avoids deleterious central side effects such as apnea and addiction. In human clinical trials and rat models of inflammatory pain, peripherally restricted opioids have repeatedly shown powerful analgesic effects; in some mouse models however, their actions remain unclear. Here, we investigated opioid receptor coupling to K^+^ channels as a mechanism to explain such discrepancies. We found that GIRK channels, major effectors for opioid signalling in the CNS, are absent from mouse peripheral sensory neurons but present in human and rat. *In vivo* transgenic expression of GIRK channels in mouse nociceptors established peripheral opioid signalling and local analgesia. We further identified a regulatory element in the rat GIRK2 gene that accounts for differential expression in rodents. Thus, GIRK channels are indispensable for peripheral opioid analgesia, and their absence in mice has profound consequences for GPCR signalling in peripheral sensory neurons.

GIRK channels are indispensable for peripheral opioid analgesia. The absence of GIRK channels from mouse dorsal root ganglion neurons questions the predictive validity of mice as a model organism for investigating peripheral GPCRmediated analgesia.

## INTRODUCTION

Opioid agonists such as morphine are the gold standard for the treatment of severe pain. Their effects are mediated by μ, δ and κ-opioid receptors that function as G protein-coupled receptors (GPCRs). Despite the widespread use of opioids to control pain, their clinical effectiveness is hampered by adverse side effects that result mainly from activation of receptors in the central nervous system (CNS). Such effects include apnea and addiction and have recently lead to an epidemic of overdoses, death and abuse (Paulozzi et al, 2012; Schumacher et al, 2009).

Opioid receptors are not only localised in the brain and spinal cord, but are also expressed in peripheral sensory neurons. A promising therapeutic approach therefore has been to develop peripherally restricted opioid analgesics that target such receptors, thus avoiding centrally mediated side effects (Stein & Machelska, 2011; Stein et al, 2003). There is considerable evidence supporting a role for peripheral opioid receptors in promoting analgesia. All three subtypes are expressed by nociceptive dorsal root ganglion (DRG) neurons that co-express markers such as TRPV1, isolectin B4 (IB4), and calcitonin gene related peptide (CGRP) (Li et al, 1998; Wang et al, 2010; Wu et al, 2008). Moreover, opioid receptors are transported to the peripheral terminals of sensory neurons in the skin (Hassan et al, 1993). Injection of opioid agonists at peripheral injury sites has been demonstrated to elicit analgesia in rat models of inflammatory and neuropathic pain (Obara et al, 2009; Stein et al, 1989) and in human clinical investigations of postoperative and arthritic pain (Kalso et al, 2002; Vadivelu et al, 2011). Of note, widely variable responses to opioids have been reported between rats and mice, and species differences have gained relevance for predictive validity since prototype compounds with demonstrated analgesic efficacy in rodents have often failed in humans (Mogil, 2009).

In view of the rising importance of mouse models and of novel gene therapeutic approaches in pain research and analgesic drug development (Goins et al, 2012; Mogil, 2009), we were intrigued by the possible discrepancy in peripheral opioid analgesia between rat and mouse. Since both species express opioid receptors on their peripheral sensory neurons (Wang et al, 2010; Wu et al, 2008), we wondered whether deficits in the coupling of receptors to downstream effectors might explain differences in opioid efficacy. In mouse CNS neurons, opioid receptors attenuate neuronal excitability via reduction of voltage gated calcium currents, inhibition of adenylyl cyclase and potentiation of potassium currents (Torrecilla et al, 2002). In mouse and rat DRG neurons, there is extensive evidence for modulation of voltage gated calcium channels (Borgland et al, 2001; Gross & Macdonald, 1987; Wu et al, 2008) and adenylyl cyclase (Makman et al, 1988). However, the role of potassium channels as opioid receptor effectors in DRG neurons has not been explored in detail.

G protein-coupled inwardly rectifying K^+^ channels (GIRK, also known as K_ir_3) are important regulators of neuronal resting potential and excitability, and are the major type of potassium channel activated by GPCRs (Luscher & Slesinger, 2010). Four different subunits (GIRK1-4) have been identified. These form either homo- or heterotetramers, but only GIRK2 is able to assemble functional homotetramers. GIRK1, -2 and -3 are all expressed in the brain where they are the primary postsynaptic effectors for GPCR signalling. Consistently, CNS GIRK channels mediate the analgesic actions of various GPCRs (opioid, M2 muscarinic, α2 adrenergic and cannabinoid receptors) and of ethanol that opens these channels directly. Interestingly, disruption of these signalling pathways in GIRK2 knockout mice has a more prominent effect in males compared to females, suggesting that GIRK channels may also be important for genetic differences in pain thresholds (Luscher & Slesinger, 2010).

Here, we investigated the role of GIRK channels as mediators of peripheral opioid analgesia in primary afferent sensory neurons. We examined the expression and function of GIRK channels in mouse, rat and human peripheral sensory neurons. Strikingly, we found no evidence of such GIRK channel expression in mouse, in contrast to human and rat. To explore the hypothesis that the absence of GIRK channels may be responsible for the lack of peripheral opioid analgesia, we generated transgenic mice expressing GIRK2 subunits in sensory neurons. Exogenous GIRK2 channels coupled robustly to opioid receptors in DRG neurons and established *in vivo* peripheral opioid mediated analgesia in these mice. Finally, we identified a distal regulatory element in the rat GIRK2 promoter that is lacking in mouse and accounts for the remarkable difference in GIRK2 expression in these species.

## RESULTS

### Absence of GIRK channel expression in mouse peripheral sensory neurons

To determine the extent of opioid receptor coupling to GIRK channels in mouse peripheral sensory neurons, we initially performed whole-cell patch clamping experiments in dissociated DRG neurons. As a first step, we established recording conditions by examining the well-described coupling of μ-opioid receptors to N-type calcium channels (Schroeder et al, 1991; Seward et al, 1991). We evoked large calcium channel currents in isolated neurons by applying a depolarising voltage step from −90 to 10 mV for 500 ms (Supporting Information Fig 1A). As has been previously reported (Borgland et al, 2001), application of the μ-opioid receptor agonist DAMGO (D-Ala^2^,*N*-MePhe^4^,glyol) decreased current amplitude and this effect was reversed by co-application of the opioid receptor antagonist naloxone (Supporting Information Fig 1A and B). Thus, opioid receptors and N-type calcium channels are present in cultured mouse DRG neurons and show robust coupling in our set-up.

**Figure 1:**
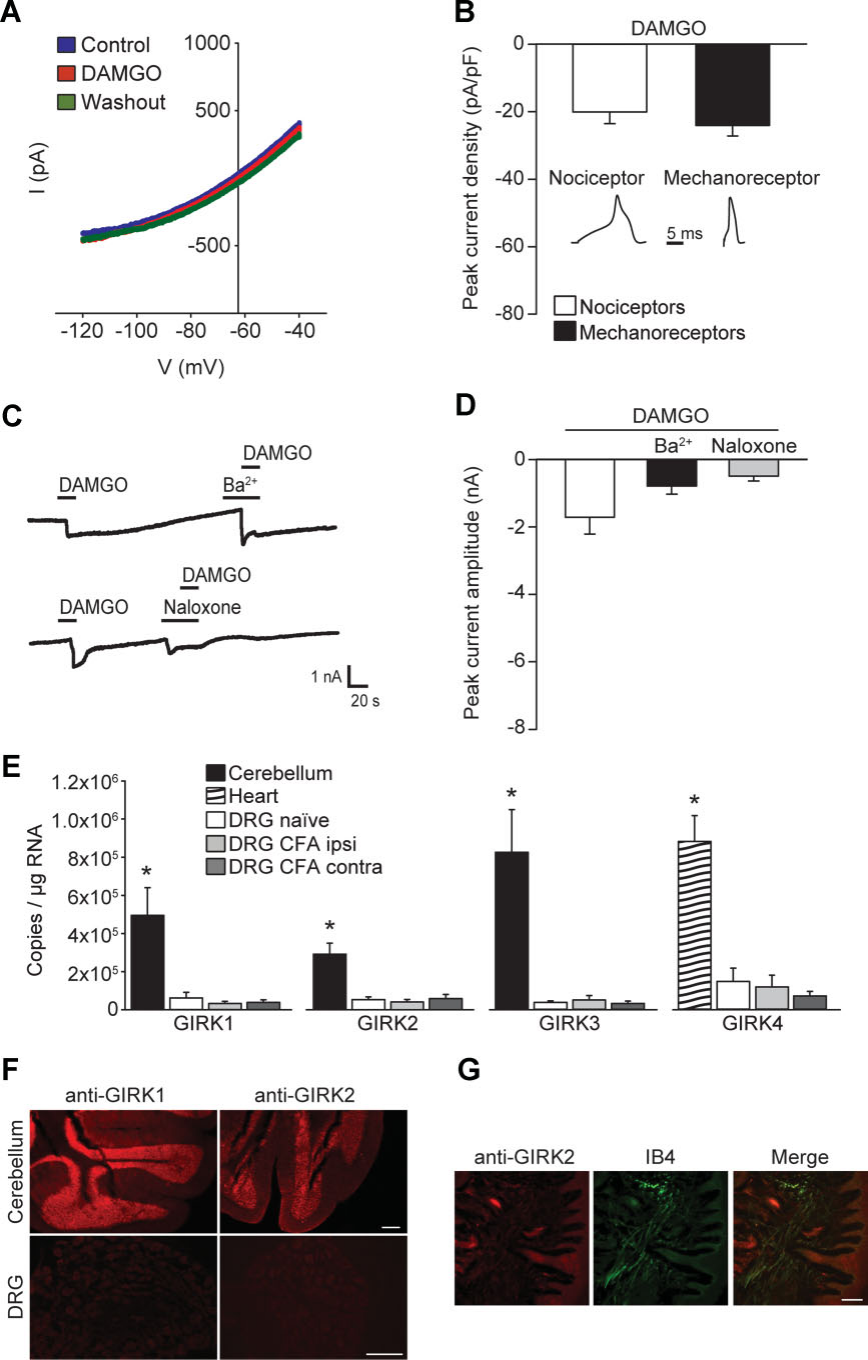
**Absence of GIRK channels in mouse DRG neurons**Traces of currents in mouse DRG neurons stimulated with hyperpolarising voltage ramps from −120 to −40 mV and treated with 10 µM DAMGO followed by washout.No differences in peak currents at −120 mV in mouse nociceptors (*n* = 28) and mechanoreceptors (*n* = 19) after 10 µM DAMGO application. Representative action potential shapes of nociceptors and mechanoreceptors (lower panel) were used to distinguish between DRG neuron subtypes (*p* = 0.237; Mann–Whitney Rank Sum test).Representative current traces of mouse DRG neurons at −80 mV after application of 10 µM DAMGO, 3 mM barium or 20 µM naloxone recorded in high potassium solution.Peak current amplitude after agonist and antagonist application (*p* = 0.066; one way repeated measures ANOVA, *n* = 8).Quantification of GIRK channel mRNA in mouse cerebellum, heart and DRG neurons from naïve animals and from animals with CFA-induced unilateral hindpaw inflammation (inflamed: CFA ipsi; noninflamed: CFA contra). (GIRK1 and GIRK2: *n* = 7; GIRK3 and GIRK4: *n* = 6; Kruskal–Wallis ANOVA on Ranks, Dunn's method compared to cerebellum or heart *p < 0.05).Sections of mouse cerebellum and DRG labeled with antibodies specific for GIRK1 and GIRK2.Mouse skin sections immunostained with anti-GIRK2 and anti-IB4. All scale bars are 50 µm.

We next assessed the contribution of GIRK channels to opioid signalling. In HEK293 cells transfected with GIRK1, GIRK2 and μ-opioid receptors, hyperpolarisation of the membrane evoked large currents that were increased by DAMGO and inhibited by tertiapin Q, a potent blocker of inward rectifier K^+^ channels (Kanjhan et al, 2005) (Supporting Information Fig 1C and D). We then applied hyperpolarising voltage ramps from −40 to −120 mV to mouse DRG neurons and recorded currents in untreated neurons, in the presence of DAMGO, and after application of tertiapin Q. From 55-recorded cells, we never observed significant inward currents or any modulation by DAMGO (Fig 1A) or tertiapin Q. We further classified these neurons into nociceptors and mechanoreceptors based upon their action potential configuration (nociceptors display broad action potentials with inflections on the falling phase, while mechanoreceptors have narrow action potentials (Stucky & Lewin, 1999; Fig 1B). Since μ-opioid receptors were shown to be expressed predominantly on nociceptors (Li et al, 1998; Scherrer et al, 2009), we reasoned that at least this subpopulation should exhibit GIRK currents. Contrary to this assumption, inward current densities evoked by DAMGO in nociceptors were minimal and indistinguishable from those in mechanoreceptors (Fig 1B). Finally, we employed a highly sensitive measure of GIRK channel activity by recording currents at −80 mV in the presence of high extracellular K^+^ concentrations. Again, application of DAMGO evoked negligible inward currents that were not significantly altered by naloxone or barium (another GIRK channel blocker that we used because tertiapin Q was ineffective in HEK293 cells transfected with GIRK2 only) (Fig 1C and D).

Taken together, these data indicate that either μ-opioid receptor coupling to GIRK channels is defective in mouse DRG neurons, or that GIRK channels are absent from cells expressing opioid receptors. To explore this further, we first inoculated mouse primary DRG neurons *in vitro* with an adeno-associated viral vector-GIRK2 construct (AAV-GIRK2). Applying voltage ramps from −40 to −120 mV, we observed large inward currents in these neurons (Supporting Information Fig 1E) indicating that mouse DRG neurons are able to support GIRK currents. We next examined the mRNA expression of GIRK1-4 subunits using quantitative RT-PCR (qRT-PCR) in mouse DRG and control tissues (Fig 1E). Since peripherally applied opioid agonists were demonstrated to have improved efficacy in inflammatory pain (Stein et al, #emmm201201980-bib-0046 [Bibr b46],[Bibr b47]), we assessed GIRK mRNA expression in lumbar DRG taken from both naïve animals and mice with unilateral paw inflammation induced by intraplantar injection of complete Freund's adjuvant. Strikingly, we observed very low expression of all GIRK channel subunit transcripts that was not altered by inflammation. In contrast, GIRK1-4 channel mRNA could be readily detected in cerebellum (Karschin et al, 1996) or heart (Wickman et al, 1997), respectively (Fig 1E).

To obtain a broader view of GIRK channel expression and determine whether sporadic cells might express channels, we immunostained mouse DRG sections with antibodies specific for GIRK1 and -2 subunits (Marker et al, 2004). We never observed GIRK1 or -2 expression in any DRG cell (although the same DRG could be readily labeled with an antibody against δ-opioid receptors, Supporting Information Fig 1F), while staining was clearly apparent in control cerebellum (Fig 1F). To exclude the possibility that GIRK channels are only detectable at peripheral nerve terminals, we also immunostained mouse skin sections with GIRK2 antibodies and the neuronal marker IB4. Again, we were unable to detect GIRK2 signal in subcutaneous nerves (Fig 1G). Finally we probed immunoblots of mouse DRG and spinal cord extracts with the GIRK1 and GIRK2 antibodies. We failed to detect GIRK subunits in DRG but observed a strong signal in immunoblots of spinal cord tissue (Supporting Information Fig 1G). Collectively, our data indicate that at the transcript, protein and functional level, GIRK channels are absent from mouse DRG, and that calcium channels may be the predominant ion channel effectors of μ-opioid receptor signalling in these neurons.

### GIRK channel expression in rat and human sensory neurons

**Figure 2:**
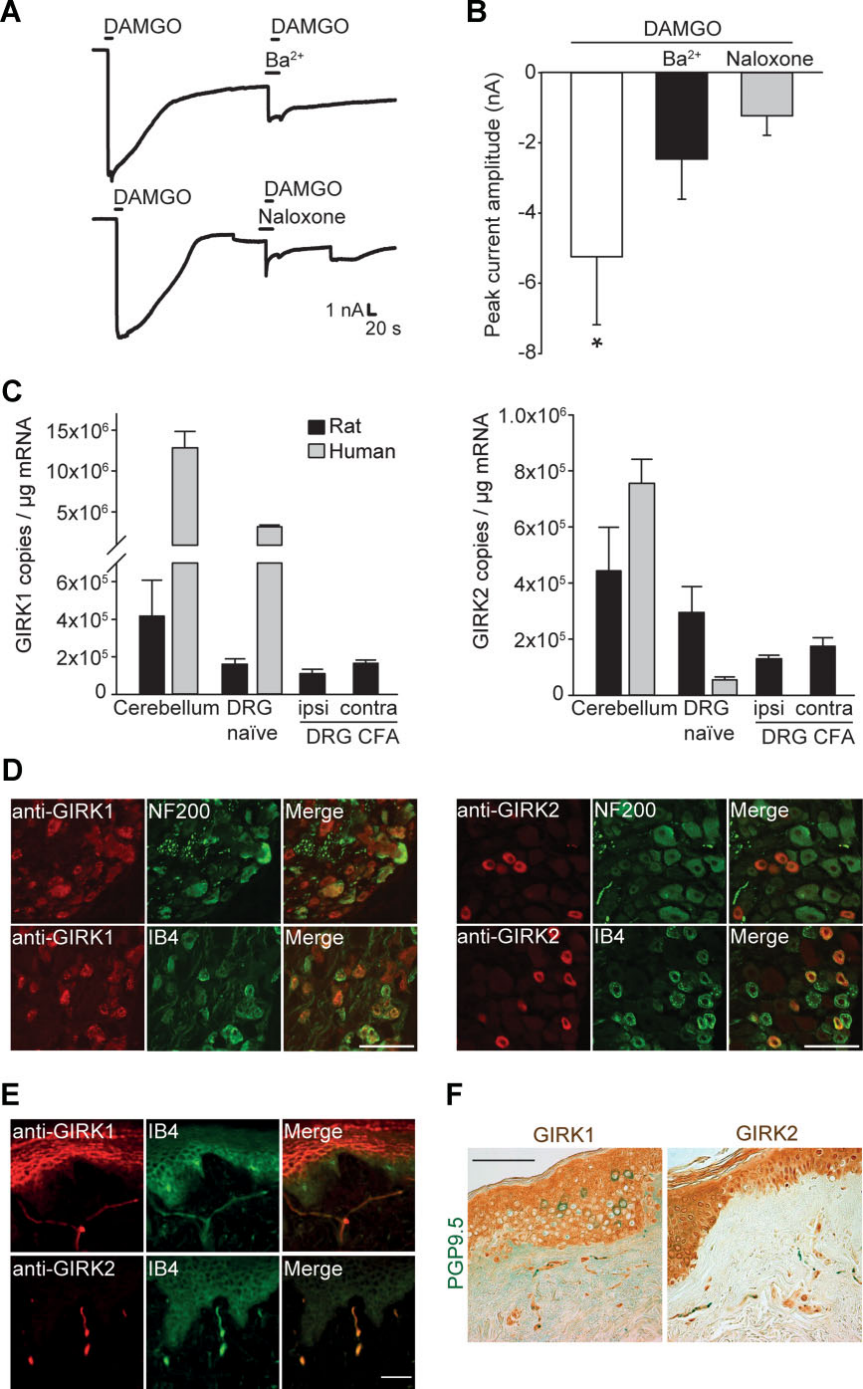
**Expression of GIRK channels in rat and human sensory neurons**Current traces of rat DRG neurons voltage-clamped at −80 mV. Inward currents were recorded in high potassium solution. Currents were evoked by 10 µM DAMGO and suppressed by GIRK channel blocker barium (3 mM) and opioid receptor antagonist naloxone (20 µM).Peak currents after agonist and antagonist application (*p = 0.037; one way repeated measures ANOVA, Holm–Sidak method, *n* = 7).Quantification of GIRK1- and -2 mRNA in rat and (postmortem) human cerebellum and naïve DRG, and in rat DRG neurons innervating the inflamed (CFA ipsi) or the non-inflamed (CFA contra) paw (*n* ≥ 3).Immunoreactivity for GIRK1 and GIRK2 in rat DRG sections. GIRK channels are detectable in nonpeptidergic IB4 positive nociceptors but not in myelinated neurons expressing NF200.Rat skin cryosections (E) and human skin paraffin sections (F) stained with antibodies specific for GIRK1, GIRK2, IB4 and PGP9.5. All scale bars are 50 µm.

To determine whether the lack of GIRK channels, we observed in mouse DRG neurons is of broad significance, we examined rat and human DRG for GIRK channel expression. We followed a similar analysis to that performed in mice and initially assessed dissociated rat sensory neurons using whole cell patch clamping. Contrary to our findings in mice, we observed robust inward currents at −80 mV in high K^+^ solution upon application of DAMGO in rat (Fig 2A and B). Currents were inhibited by the GIRK channel blocker barium and by the opioid receptor antagonist naloxone (Fig 2A and B), indicating that GIRK channels are present in rat DRG and that they couple to opioid receptors.

We examined GIRK channel expression further at the transcript level using qRT-PCR from rat and human DRG. We were able to detect mRNA of GIRK channel subunits in both rat and human DRG at levels significantly higher than those observed in mouse (Fig 2C). There were, however, differences in subunit expression across species, with GIRK1 mRNA being the prominent transcript in human and GIRK2 mRNA in rat DRG (Fig 2C). We observed no increase in GIRK expression in rat DRG under inflammatory pain conditions (Fig 2C).

To investigate the distribution of GIRK subunits in rat DRG, we performed immunostaining and detected immunoreactivity of both GIRK1 and GIRK2 (Fig 2D). We explored the identity of GIRK subunit expressing cells by co-staining sections with an antibody against Neurofilament 200 (NF200), a marker for myelinated neurons (Perry et al, 1991), or IB4, a marker of non-peptidergic nociceptors (Stucky & Lewin, 1999). GIRK1 or GIRK2 subunits were rarely co-localised with NF200, whereas both subunits frequently coincided with IB4 (Fig 2D). Thus, GIRK channels are expressed predominantly by nociceptors in rat DRG.

To determine whether GIRK1 and -2 are transported to the peripheral terminals of sensory neurons, we carried out immunostaining of rat and human skin sections. Both GIRK1 and -2 immunoreactivity was clearly discernible in rat and human skin, and in addition to expression in keratinocytes coincided with IB4 or PGP9.5 positive neurons innervating the dermis (Fig 2E and F). Taken together, our data demonstrate that GIRK channels are expressed by human and rat peripheral sensory neurons and couple with opioid receptors in the rat, suggesting that they may be important effectors for opioid signalling in DRG neurons from these species.

### Expression of functional GIRK2 channels in sensory neurons of a Na_v_1.8-GIRK2 transgenic mouse

We next sought to confirm that the absence of GIRK channels in DRG neurons indeed underlies the lack of antinociceptive effects of peripherally applied opioids observed in some mouse models. We reasoned that by expressing GIRK channels exogenously in mouse DRG *in vivo*, we would be able to explore their relative contribution to opioid receptor signalling and ultimately determine whether they are able to establish peripheral opioid analgesia in mice.

Since GIRK2 is the only subunit that assembles into functional homo-tetrameric channels (Luscher & Slesinger, 2010), we generated transgenic mice expressing a FLAG-GIRK2 construct under the regulatory elements of the sodium channel Na_v_1.8 to drive selective expression in peripheral sensory neurons (Shields et al, 2012). We identified one transgenic founder line and, using Southern blot and qPCR, detected that nine copies of the transgene had integrated into the genome (Supplementary Fig 2A). These mice are referred to as Na_V_1.8-GIRK2 mice.

**Figure 3:**
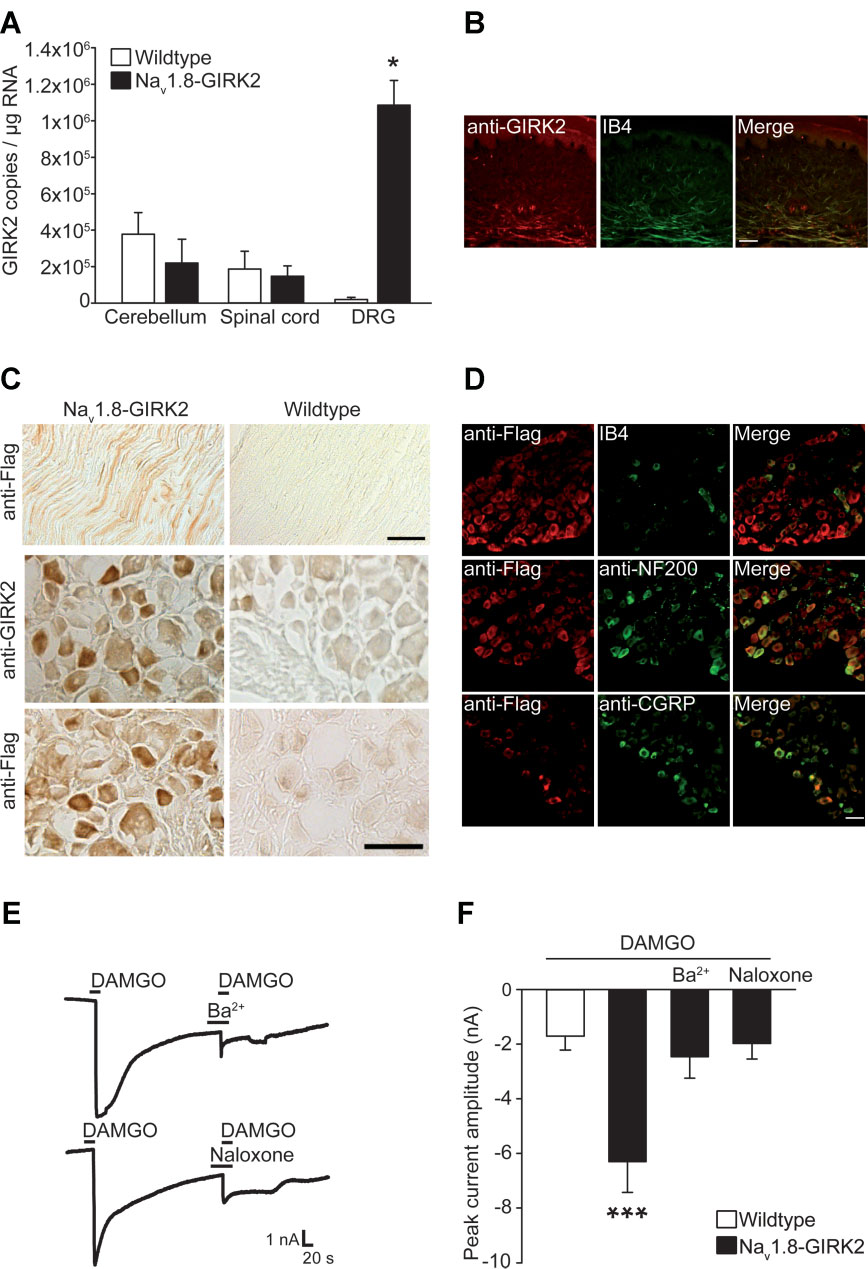
**Expression of functional GIRK2 channels in DRG neurons of Nav1.8-GIRK2 transgenic mice**GIRK2 mRNA expression in cerebellum, spinal cord and DRG neurons of Nav1.8-GIRK2 and wildtype mice (*p = 0.05, One-way ANOVA, Holm–Sidak method, *n* = 3).Skin sections from Nav1.8-GIRK2 mice labeled with antibodies specific for GIRK2 and IB4.Immunoreactivity for FLAG and GIRK2 demonstrating the expression of exogenous Flag-GIRK2 in sciatic nerve (uppermost panel) and DRG neurons (lower panels) in Nav1.8-GIRK2 mice.Sections of DRGs from Nav1.8-GIRK2 mice immunostained with anti-FLAG and co-stained with IB4, anti-NF200 and anti-CGRP. All scale bars are 50 µm.Representative current traces of Nav1.8-GIRK2 DRG neurons recorded in high potassium solution. Neurons voltage-clamped at −80 mV showing large inward currents evoked by 10 µM DAMGO and inhibited by barium (3 mM) and naloxone (20 µM).Peak current amplitude after application of agonists and antagonists in wildtype and Nav1.8-GIRK2 DRG neurons (***p = 0.001, two way ANOVA, Holm–Sidak method, *n* = 9–12 cells per group). All scale bars are 50 µm (B–D).

We first examined the expression of GIRK2 mRNA in transgenic mice using qRT-PCR. Similar levels of mRNA were present in cerebellum and spinal cord of Na_V_1.8-GIRK2 mice and wildtype littermates indicating that the transgene was not ectopically targeted to these tissues (Fig 3A). In contrast, we observed high levels of GIRK2 transcript in DRG of Na_v_1.8-GIRK2 mice confirming that the Na_v_1.8 promoter drives expression in sensory neurons (Fig 3A). We observed no alterations in GIRK2 mRNA levels following intraplantar injection of CFA to model inflammatory pain, or after chronic constriction injury of the sciatic nerve to model neuropathic pain (Supporting Information Fig 2B).

We next explored the expression of GIRK2 protein in Na_v_1.8-GIRK2 mice by immunostaining sections of skin, nerve, and DRG with antibodies against GIRK2 and the FLAG epitope. We detected robust expression of GIRK2 in peripheral terminals of sensory neurons in the skin (Fig 3B) and in axons of the sciatic nerve (Fig 3C). GIRK2 and FLAG staining was also evident in neuronal soma of the DRG with marked expression in many neurons (Fig 3C). We performed fluorescent immunocytochemistry on dissociated DRG to identify these neurons more explicitly. GIRK2 and FLAG immunoreactivity consistently overlapped in DRG neurons, indicating that both antibodies recognised the transgene protein product (Supporting Information Fig 2C). FLAG immunoreactivity also co-localised to some extent with CGRP, a marker of peptidergic nociceptors (Li et al, 1998), with the non-peptidergic nociceptor marker IB4 (Stucky & Lewin, 1999) and with the μ-opioid receptor (Supporting Information Fig 2C). Finally, we also performed fluorescent immunohistochemistry on DRG sections. We observed strong FLAG immunoreactivity across many neurons that were positive for nociceptive markers IB4, and CGRP (Fig 3D). We also detected some staining of the FLAG-epitope in NF200 positive neurons indicating that transgene expression is not limited to unmyelinated neurons (Fig 3D).

We then investigated the coupling of the transgenic GIRK2-FLAG with opioid receptors using whole cell patch clamping and K^+^ imaging. We observed no difference in the resting membrane potential of neurons from Na_v_1.8-GIRK2 mice compared to wildtype littermates (−51.9 ± 2.8 mV versus −51.4 ± 3 mV respectively). However, analogous to our findings in rat, application of DAMGO evoked large inward currents in DRG neurons from transgenic mice that were inhibited by barium and by naloxone (Fig 3E and F). We explored this further utilising the thallium-sensitive dye FluxOR to quantify the flux of thallium ions through K^+^ channels as a measure of GIRK channel activity (Beacham et al, 2010). In neurons isolated from Na_v_1.8-GIRK2 mice, we observed a significantly higher uptake of thallium ions upon application of DAMGO compared to wildtype littermates (Supporting Information Fig 2D and E). A larger thallium influx was also detected after application of another opioid agonist (DPDPE; data not shown), while no difference in uptake was observed in the absence of opioids (Supporting Information Fig 2E). Thus, exogenously expressed GIRK2 in mouse sensory neurons *in vivo* couples to opioid receptors and reproduces the phenotype we described in rat DRG neurons.

### Exogenous GIRK2 expression in mouse peripheral sensory neurons establishes peripheral opioid mediated analgesia

**Figure 4:**
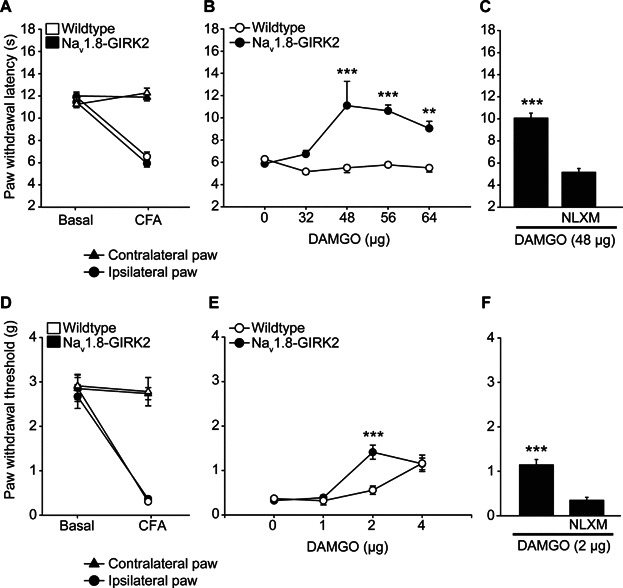
**Antinociceptive effect of peripherally applied DAMGO in Nav1.8-GIRK2 mice**Changes in paw withdrawal latency in response to radiant heat in the inflamed (ipsilateral) and noninflamed (contralateral) paws of Nav1.8-GIRK2 and wildtype mice.Dose-dependent inhibition of thermal hyperalgesia 5 min after DAMGO injection into the inflamed paw of Nav1.8-GIRK2 mice (unadjusted *p*-values: ***p = 0.000, **p = 0.004; two way ANOVA, Holm–Sidak method, *n* = 7–8).DAMGO-induced antinociception is reversed by co-injection of naloxone-methiodide (NLXM, 5µg) in the inflamed paw of Nav1.8-GIRK2 mice (***p < 0.001; Student's unpaired *t*-test, *n* = 7–8).Changes in paw withdrawal threshold in response to mechanical stimuli upon induction of inflammation (ipsilateral) compared to the noninflamed (contralateral) side in Nav1.8-GIRK2 and wildtype mice.Elevation of paw withdrawal threshold after injection of 2 µg DAMGO into the inflamed paw of Nav1.8-GIRK2 mice (***p < 0.001; two-way ANOVA, Holm–Sidak method, *n* = 8).DAMGO (2 µg)-induced antinociception is reversed by co-injection of NLXM (5 µg) in the inflamed paw of Nav1.8-GIRK2 mice (***p < 0.001; Student's unpaired *t*-test, *n* = 8).

We reasoned that Na_v_1.8-GIRK2 transgenic mice would allow us to assess the importance of GIRK channels for pain and peripheral opioid mediated analgesia *in vivo*. To quantify nociceptive behaviour we measured hindpaw withdrawal responses to thermal and mechanical stimuli under baseline conditions and following intraplantar injection of CFA to model inflammatory pain. Sensitivities to both a radiant heat source and to calibrated von Frey filaments were indistinguishable between Na_v_1.8-GIRK2 mice and wildtype littermates (Fig 4A and D). Similarly, both Na_v_1.8-GIRK2 and control mice developed pronounced hyperalgesia to thermal and mechanical stimuli after CFA injection that was essentially identical (Fig 4A and D). Thus, exogenous expression of GIRK2 in mouse peripheral sensory neurons does not modulate basal nociceptive thresholds.

We next asked whether subcutaneous application of DAMGO into the hindpaw of Na_v_1.8-GIRK2 transgenic mice would reveal a role of GIRK channel-opioid receptor coupling in antinociception. Local injection of DAMGO had no effect on thermal response latencies in either Na_v_1.8-GIRK2 or wildtype mice without inflammation (Supporting Information Fig 2F). In wildtype mice with paw inflammation, DAMGO did not significantly reduce thermal or mechanical hyperalgesia either (Fig 4B and E). Strikingly however, in Na_v_1.8-GIRK2 mice with inflammatory pain, application of DAMGO to the hindpaw dose-dependently inhibited the development of thermal and mechanical hyperalgesia (Fig 4B and E). Of note, these effects peaked at 48 and 2 μg DAMGO, respectively, and were strongest for thermal stimuli, where baseline response latencies were fully reinstated (Fig 4B).

We also tested whether the antinociceptive actions of DAMGO in Na_v_1.8-GIRK2 mice were indeed occurring at a peripheral site. We co-injected DAMGO and naloxone-methiodide (NLXM), an opioid receptor antagonist that does not cross the blood–brain barrier, and measured behavioural responses. NLXM strongly inhibited the analgesic effects of DAMGO on both thermal and mechanical withdrawal thresholds and reduced levels to those observed in wildtype mice (Fig 4C and F). Finally, as a reference, we reexamined the effects of DAMGO on CFA induced nociceptive behaviour in rats. As previously reported (Stein et al, 1989), DAMGO dose dependently reversed thermal and mechanical hyperalgesia in rats (albeit at lower concentrations than in Na_v_1.8-GIRK2 mice), an effect that was also reversed by co-application of NLXM (Supporting Information Fig 3).

Collectively, our data indicate that exogenous expression of GIRK2 *in vivo* does not influence normal nociceptive behaviour, however, activation of these channels via μ-opioid receptor signalling is sufficient to restore peripheral opioid mediated analgesia in mice.

### Regulation of GIRK2 gene expression in rodent sensory neurons

In light of the striking difference in expression of GIRK channels in DRG neurons of mice versus rats, we sought to determine the mechanisms that regulate GIRK gene expression. We focused initially on GIRK2 because this subunit was expressed in rat but not mouse nociceptors and the high level of sequence conservation between these species could allow for a more definitive analysis of the structural elements that govern its transcription.

**Figure 5:**
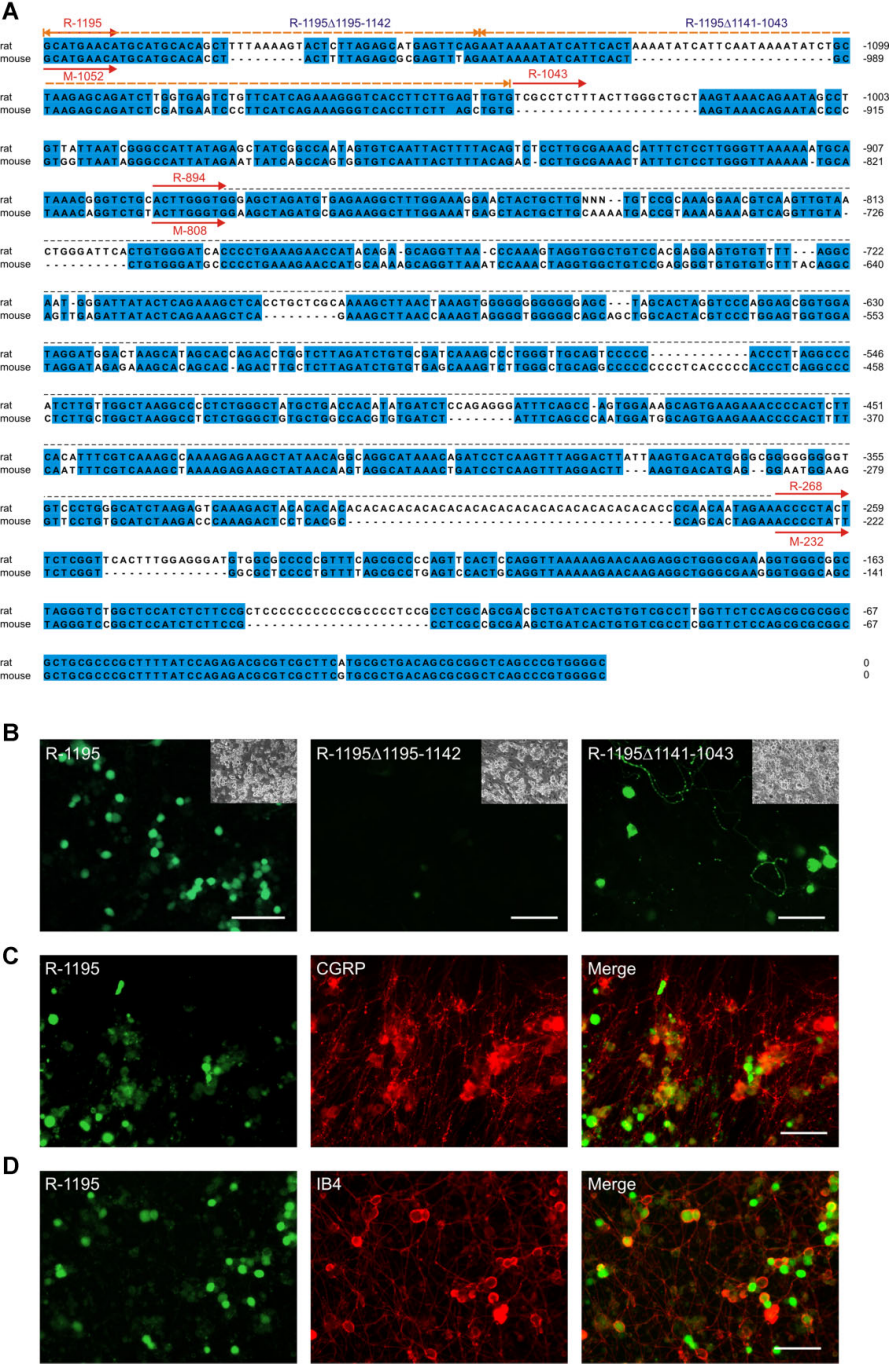
**Identification of a regulatory sequence in the rat *Kcnj6* gene that drives expression in peripheral sensory neurons**Sequence alignment of rat and mouse *Kcnj6* upstream of the transcription start-site. Dotted black line indicates sequence that was absent from public databases. Dotted orange lines designate regions that were deleted from the R-1195 reporter construct.eGFP fluorescence in mouse DRG cultures transfected with R-1195-eGFP, R-1195Δ1195–1142-eGFP and R-1195Δ1141–1043-eGFP reporter constructs. Insets show phase contrast images.Co-staining of R-1195-eGFP transfected sensory neurons with anti-CGRP.Co-staining of R-1195-eGFP with IB4. All scale bars are 100 μm.

As a starting point, we constructed a sequence alignment of the region upstream of the transcription start-site of the rat and mouse GIRK2 gene (*Kcnj6*). Mouse genomic sequence was well annotated and readily available from public databases. In contrast, the rat promoter displayed poor coverage, especially prior to position −267 relative to the transcription start-site (indicated in Fig 5A). We thus sequenced this region from rat genomic DNA and obtained full coverage which aligned well with the equivalent mouse sequence.

From the alignment, it is apparent that there are a number of short sequence differences between rat and mouse that could function to regulate selective GIRK2 expression. Indeed, the rat genomic sequence was found to contain three larger (20–30 bp) insertions embedded in fully conserved regions (murine) that might be responsible for the striking difference of gene expression among species. We divided the 1kb upstream regulatory region into several shorter sections and generated constructs consisting of potential promoter upstream elements fused to a promoterless eGFP reporter plasmid (Fig 5A).

We reasoned that transfection of the reporter constructs into dissociated mouse DRG would drive differential eGFP expression in neurons if any of the insertions functioned as regulatory elements in these cells. As a positive control we first transfected an eGFP plasmid containing a strong human cytomegalovirus (CMV) promoter. We observed robust fluorescence in many neuronal and non-neuronal cells demonstrating efficient transfection (data not shown). We next transfected the mouse reporter constructs under identical conditions, and from three separate experiments never detected eGFP fluorescence for any of the constructs (Supporting Information Fig 4A). Likewise, the reporters comprising the three most proximal regions to the rat transcription start-site were insufficient to drive eGFP expression (Supporting Information Fig 4B). Strikingly however, transfection of the rat reporter plasmid containing the longest upstream promoter fragment (R-1195) induced strong eGFP fluorescence in mouse DRG cells that was localised exclusively to neurons (Fig 5B). We also examined the expression of all reporter constructs in cultures of cerebellum (Supporting Information Fig 5) and cortex (data not shown). We were not able to detect fluorescence in any cells from these cultures indicating that further brain specific regulatory elements must be present in the *Kcnj6* gene outside of the region we examined.

There are two insertions in the rat sequence that could account for the DRG-specific expression of R-1195, and considering flanking sequences, both of them would have the appropriate size to serve as a transcription factor-binding site (annotated in Fig 5A). We investigated this further by generating reporter constructs consisting of R-1195 with deletions of either of these regions (R-1195Δ1195–1142 and R-1195Δ1141–1043, Fig 5A). Removal of the smaller insertion more distal to the transcription start site (R-1195Δ1195–1142) led to a dramatic reduction in the number of eGFP positive DRG neurons and only very sparse expression was observed (Fig 5B). Deletion of the longer insertion (R-1195Δ1141–1043) reduced eGFP expression but to a lesser extent than R-1195Δ1195–1142 (Fig 5B). These data indicate that both motifs are able to drive expression in DRG neurons and may contain recognition sites for DRG specific transcription factors that act cooperatively to regulate gene expression. However, the observation that deletion of the distal region has a stronger effect suggests that this is the prominent sequence for regulation of GIRK2 expression in rat DRG.

To determine whether R-1195-induced fluorescence localises to nociceptors, we also co-stained DRG cultures with the markers CGRP and IB4. R-1195-eGFP fluorescence was prominent in small sized neurons and displayed some overlap with CGRP and IB4 (Fig 5C and D). Thus, the differences in the rat and mouse promoter region that we identified here may account for specific GIRK2 expression in rat nociceptors.

We further explored whether the absence of GIRK2 expression in mouse DRG occurs as a result of inbreeding in laboratory mouse strains. In the current study, we performed all experiments on the C57BL6 strain, and the sequence information for the mouse *Kcnj6* gene was also derived from C57BL6. We obtained sequences of 17 mouse genomes from the Mouse Genomes Project (Keane et al, 2011) (http://www.sanger.ac.uk/resources/mouse/genomes/), of which 10 displayed high quality coverage of the *Kcnj6* locus on chromosome 16. Alignment of these sequences in the region proximal to the transcription start site revealed a high level of similarity to the C57BL6 reference genome, even in the wild-derived inbred strains CAST/EiJ, PWK/PhJ, WSB/EiJ and SPRET/EiJ (Supporting Information Fig 6). Of note, the putative DRG localisation signals we identified in rat are lacking from all mouse sequences, and indeed only two smaller insertions more proximal to the transcription start-site are conserved between rat and the outbred SPRET/EiJ strain. We thus conclude that the remarkable differences in regulation of rat versus mouse expression of GIRK2 in nociceptors are evolutionarily conserved and presumably reflect adaptations of mouse or rat to their natural habitats.

Finally, we applied a similar analysis to the human *KCNJ6* (GIRK2) gene and to a region 2100 bp upstream of mouse and human orthologues of *KCNJ3* (GIRK1, see Supplementary Methods for details). None of these reporter constructs were sufficient to drive eGFP expression in mouse DRG neurons indicating that homologous regulatory regions for the human genes reside outside of the area considered here.

## DISCUSSION

GPCRs such as opioid receptors are the largest family of membrane receptors and represent major targets for drug discovery. Here, we have investigated an apparent species difference in GPCR signalling that impacts upon the effectiveness of opioid analgesia in mouse versus rat models of inflammatory pain, and on the predictive validity of such models for human medicine. We demonstrate that GIRK channels, primary effectors for GPCRs in the CNS, are absent from mouse peripheral sensory neurons but are expressed in rat and human. We further show that functional GIRK channels are sufficient for peripheral opioid-mediated analgesia *in vivo*, and identify a short regulatory region distal to the promoter of the rat *Kcnj6* gene that accounts for expression in rat sensory neurons.

Potential species differences in the efficacy of peripherally applied opioids have not been explored extensively. There is considerable evidence for a peripheral effect of opioids in rats (Obara et al, 2009; Stein et al, #emmm201201980-bib-0046 [Bibr b46],[Bibr b47]) and humans (Kalso et al, 2002; Vadivelu et al, 2011), where local application of opioid agonists is antinociceptive under a number of different inflammatory pain conditions. Similar to our experiments (Fig 4; Supporting Information Fig 3), other studies on peripheral opioid analgesia in mice have often reported higher doses of opioids required to achieve effectiveness similar to rats (Boue et al, 2012; Cunha et al, 2010; Hernandez et al, 2009; Obara et al, 2009; Stein et al, 1989), despite the presence of opioid receptors in mouse DRG neurons (Scherrer et al, 2009; Wang et al, 2010). Our analysis suggests that there are significant differences in opioid receptor signalling between rat and mouse sensory neurons, and that these differences may contribute to the mechanisms that determine species-specific expression of peripheral opioid analgesia.

To explore potential mechanisms, we focused on a lack of K^+^ channel modulation by opioid receptors. In support of our findings, it has been reported that mouse sensory neurons lack opioid-sensitive K^+^ currents at resting and hyperpolarised membrane potentials (Kanjhan et al, 2005), and that GIRK2 immunoreactivity is absent from mouse sensory neurons (Mitrovic et al, 2003). Furthermore, a number of studies have demonstrated that GIRK channels are present in rat DRG neurons at the transcript (Gao et al, 2007), protein (Khodorova et al, 2003) and functional level (Gao et al, 2007). We therefore suggest that wildtype mice can be regarded as sensory neuron-specific ‘knockouts’ for GIRK channels and thus represent a valuable tool for examining the role of GIRK channels in peripheral opioid analgesia and other neuronal functions.

It is surprising that we observed no difference in the resting membrane potential of sensory neurons from Na_v_1.8-GIRK2 mice, as in the CNS, deletion of GIRK2 depolarises the membrane potential in the absence of receptor stimulation (Luscher et al, 1997). Moreover, because baseline nociceptive thresholds were essentially normal in transgenic mice, our data imply that there is no basal GIRK activity in DRG neurons from Na_v_1.8-GIRK2 mice, and that constitutive or endogenous GPCR activation does not impinge upon GIRK channels in these mice.

Our data accords well with observations made in rat where peripherally active opioids are often ineffective if applied to healthy tissue but elicit pronounced antinociception upon induction of inflammation (Stein & Machelska, 2011; Stein et al, 1989). A number of factors could contribute to this phenomenon including upregulation of opioid receptors, enhanced G-protein coupling of receptors, facilitated access of opioid ligands to their receptors (reviewed in (Stein & Machelska, 2011)), and increased GIRK channel activity during inflammation (Gonzalez-Rodriguez et al, 2010). We observed no differences in GIRK subunit mRNA expression in models of inflammatory and neuropathic pain. However, increased activity could result from posttranslational regulation such as augmented trafficking of channels to the plasma membrane (Chung et al, 2009), and potentiation by other intracellular and extracellular factors (Han et al, 2003; Mao et al, 2002).

Intriguingly, we observed differences in the sensitivity of Na_v_1.8-GIRK2 mice to the μ-opioid agonist DAMGO in thermal versus mechanical hyperalgesia. One possible explanation for this discrepancy concerns the functional organisation of DRG neurons. It has recently been demonstrated that different subsets of nociceptive neurons mediate responses to noxious thermal versus mechanical stimuli (Cavanaugh et al, 2009). It is unlikely that the Na_v_1.8-GIRK2 transgene is expressed mainly in thermally versus mechanically responsive neurons, since the Na_v_1.8 promoter drives expression across all subtypes of peripheral sensory neurons (Shields et al, 2012), and we observed expression of GIRK2 in both CGRP- and IB4-positive neurons. Instead, coupling of GIRK channels to opioid receptors, or expression of the receptor itself may underlie these differences. Indeed, it has been proposed that μ-opioid receptors are located predominantly on peptidergic neurons that mediate heat pain, while δ-opioid receptors are expressed by non-peptidergic neurons and control mechanical pain in mice (Scherrer et al, 2009). Another study showed enhanced sensitivity to peripheral opioids in mechanical versus thermal hyperalgesia in a mouse model of neuropathic pain (Gaveriaux-Ruff et al, 2011). Thus, the enhanced sensitivity of thermal responses to peripherally applied DAMGO reported here could occur because μ-opioid receptors couple to GIRK2 channels principally in heat responsive neurons. These issues need to be clarified in future experiments.

Interspecies variability and genetic influences on pain sensitivity are a major research focus (Mogil, 2009). Many studies have examined inbred mouse strains and discovered a strong genetic component for systemic morphine analgesia (Belknap et al, 1998; Elmer et al, 1998; Mogil, 2009). A number of loci have been demonstrated to contribute to this variability, and interestingly, the GIRK3 gene *Kcnj9* has been implicated as a candidate gene affecting opioid analgesia (Smith et al, 2008). In addition, polymorphisms in the GIRK2 gene *KCNJ6* have been associated with increased opioid requirements in human patients after abdominal surgery. In both studies, *cis*-acting genetic elements in these genes were shown to regulate expression of GIRK channels in different brain regions. Here we defined two insertions upstream of the distal promoter of rat *Kcnj6* that drive expression of GIRK2 exclusively in rat and not mouse DRG neurons. Because of a low level of conservation between mouse and human sequences in the *KCNJ3* (GIRK1) and *KCNJ6* genes, we were unable to identify regulatory regions for human GIRK channels. However, identification of *cis*-acting elements that drive GIRK channel expression in human sensory neurons, and elucidation of the transcriptional network that regulates expression of these genes, holds much promise for future research.

In summary, we have shown that GIRK channels in DRG neurons are essential for the manifestation of peripheral opioid-mediated analgesia. We describe a species difference in the expression of GIRK channels in rodent sensory neurons that we propose underlies the differential sensitivity to peripherally applied opioids. Our findings could have profound consequences for the examination of dependence and tolerance occurring at peripheral opioid receptors, and for the discovery and evaluation of novel analgesics that target other peripheral GPCRs (Luscher & Slesinger, 2010; Stein & Machelska, 2011). The absence of GIRK channels, a major signalling effector for GPCRs, in mouse DRG, raises concerns over the predictive validity of mice as a model organism for investigating peripheral GPCR-mediated analgesia. The Na_v_1.8-GIRK2 transgenic mouse described here, or humanisation of mouse GIRK genes *in vivo* would be a first step in addressing such issues.

## MATERIALS AND METHODS

### Pharmacological reagents and chemicals

DAMGO and DPDPE were purchased from Bachem, Tertiapin-Q from Alomone. All other chemicals were purchased from Sigma if not otherwise stated in the text.

### Electrophysiology

Whole cell patch clamp recordings were made on dissociated DRG neurons 12–24 h after plating or on HEK293 cells transfected with plasmid DNA encoding GIRK1, GIRK2 and the µ-opioid receptor. Gigaseals were formed with glass electrodes with a resistance between 3 and 5 MΩ and filled with intracellular solution containing (mM): 122 KCl, 11 EGTA, 1 CaCl_2_, 2 MgCl_2_, 5 NaCl, 10 HEPES, 4 MgATP, 0.25 NaGTP, pH 7.25. Currents were recorded under voltage-clamp conditions using whole-cell patch-clamp configuration with an EPC-10 amplifier (HEKA Electronic) and analyzed using Pulse software (HEKA). Data were sampled at 10-20 kHz and filtered at 2-5 kHz. Capacitance was continuously monitored and compensated using the auto function of Pulse.

For cells stimulated with hyperpolarising voltage ramps, gigaseals were formed in a bath solution containing (mM): 150 NaCl, 5 KCl, 1 CaCl_2_, 1 MgCl_2_, 10 HEPES, 10 D-Glucose, pH 7.4. To induce action potentials the amplifier was switched to current-clamp mode and currents were injected from 0.02 to 10 nA for 80 ms.

Recordings at −80 mV were performed as described elsewhere (Raveh et al, 2010). Briefly, gigaseals were formed in low K^+^ bath solution containing (mM): 140 NaCl, 5.6 KCl, 2.6 CaCl_2_, 1.2 MgCl_2_, 10 HEPES, 2.6 D-Glucose, pH 7.4. Afterwards the bath solution was changed to high K^+^ solution comprising (mM): 140 KCl, 2.6 CaCl_2_, 1.2 MgCl_2_, 5 HEPES, pH 7.4. Compounds were applied sequentially in the following order; DAMGO, DAMGO plus Nalaxone, washout, DAMGO, DAMGO plus Barium. We observed minimal desensitisation to repeated DAMGO applications (Supporting Information Fig 1H).

In experiments performed on DRG from Na_v_1.8-GIRK2 mice, neurons were selected blindly and data was pooled for statistical analysis.

For whole cell calcium channel current recordings cells were stimulated by voltage steps to +10 mV from a holding potential of −90 mV for 500 ms in a bath solution containing (mM): 140 NaCl, 5.4 CsCl, 10.8 BaCl_2_, 1 MgCl_2_, 10 D-Glucose, 10 HEPES, pH 7.3 with CsOH. The recording electrodes were filled with (mM): 100 CsCl, 5 MgCl_2_, 10 EGTA, 2 ATP, 0.25 cAMP, 40 HEPES, pH7.3 with CsOH.

### Quantitative qRT- PCR

Total RNA was isolated using RNeasy-mini-kit (Qiagen) or, in case of human RNA, purchased from Clontech Laboratories, Inc. 0.1–1 µg of total RNA was reverse transcribed utilising AMV-reverse transcriptase (Shekarabi et al, 2012). Quantitative real time PCR was performed utilising the LightCycler 1.5 instrument (Shekarabi et al, 2012) or the Mastercycler® ep *realplex* (Eppendorf). Copy numbers were calculated by serial dilutions of GIRK1 and GIRK2 plasmid DNA or dilutions of PCR products from control tissue. Assays were done with the Fast start DNA Master SYBR Green I assay (Shekarabi et al, 2012). All experiments were performed in duplicates or triplicates. The samples were analyzed by melting curves and sequenced once after amplification.

### Cell culture

Native cells were grown at 37°C in 5% CO_2_ in a cell incubator. HEK293 cells were maintained in Dulbecco's Modified Eagle Medium (GIBCO) supplemented with 10% foetal bovine serum (Biochrom AG) and 1% penicillin/streptomycin (Biochrom AG) and transfected using FuGENE®HD (Shekarabi et al, 2012). The cells were trypsinised and used for electrophysiology 12 h after transfection. Mouse and rat DRG neurons were cultured using protocols described before (Caspani et al, 2009; Endres-Becker et al, 2007).

Adeno-associated viral vector was produced as described previously (Huser et al, 2002). Dissociated DRG neurons were transduced with a GIRK2-AAV vector by incubation of the cells with 1 × 10^9^ virus particles for 45 min. Afterwards DRG medium was added to the cells and incubation was continued for another 24 h. The next day the medium was changed and the cells were maintained for 5 days before patch clamp experiments were performed.

### Immunohistochemistry

Tissues of adult rats and mice were fixed by transcardial perfusion with 4% paraformaldehyde (PFA), postfixed, cryoprotected, embedded in OCT compound (Miles Inc.), frozen and sectioned by cryostat. DRGs, sciatic nerves and skin were sectioned in 10–12 µm, spinal cord in 14 µm and the cerebellum was stained as floating section with a thickness of 40 µm. Human skin 20 µm paraffin sections were rehydrated in xylene and ethanol before stainings.

### Immunofluorescence

Sections were blocked in 5% normal goat serum (Vector) in PBS^+^ (containing PBS, 0.3% Triton X-100 and 1% BSA), then incubated overnight at 4°C with the following primary antibodies diluted in PBS^+^ and 2% normal goat serum: anti-GIRK1 (Alomone; 1:200) or anti-GIRK2 (Alomone; 1:200) or anti-GIRK2 (Chemicon; 1:500) or anti-FLAG (1:500) or anti-δ opioid receptor (Gramsch; 1:800) or CGRP (Santa Cruz; 1:25) and NF200 (1:800) or with FITC-labeled IB4 (Vector; 1:200). After washes, tissues were incubated for 2 h at room temperature with fluorescent secondary antibodies conjugated with FITC (goat anti-chicken, 1:200; Vector Laboratories) or Texas red (goat anti-rabbit, 1:200; Vector Laboratories).

### Human skin staining of GIRK channels and PGP9.5

Human skin tissue from healthy donors was kindly provided by L. Ehler from the department of dermatology, university hospital bonn, Germany. Sections prepared from routine biopsies were treated with 0.3% H_2_O_2_ in PBS^+^ for 30 min and washed with PBS. After blocking with 5% normal goat serum in PBS^+^, sections were incubated with primary antibody (concentrations as above) overnight at 4°C and then stained with diaminobenzidine (DAB), using a biotinylated secondary antibody and an avidin-biotin peroxidase complex according to the manufacturer's instructions (VECTASTAIN Elite Kit; Vector Laboratories). After enzymatic reaction sections were treated with 3% H_2_O_2_ in PBS for 30 min and washed. After blocking with 5% normal goat serum in PBS^+^, sections were incubated with primary rabbit anti human PGP9.5 antibody (Dako, 1:50) for 1 h at 30°C and overnight at 4°C, then stained with Histogreen as a substrate, using biotinylated secondary antibody and an avidin-biotin peroxidase complex (as above). The chromogen DAB used for the primary antiserum (GIRK) appeared brown, the Histogreen used for the second antiserum (PGP9.5) appeared green. After the second enzymatic reaction, sections were washed, dehydrated, cleared and mounted as above. All stainings were viewed and photographed under a microscope (Zeiss) with appropriate filters. All experiments were also performed without the first antibody to confirm their specificity.

### Immunocytochemistry

Cultured DRG neurons were fixed with 4% PFA and 4% sucrose in PBS, permeabilised with 0.25% Triton X-100 and blocked with 10% BSA in PBS. The cells were incubated with the following antibodies diluted in 3% BSA in PBS for 2 h at 37°C. Anti-GIRK2 (Alomone), anti-FLAG M2, anti-CGRP (Biomol) and anti-μ opioid receptor (Abcam) were used at 1:1000, FITC-labeled IB4 (Vector) at 1:500. Secondary antibodies conjugated to Texas red (goat anti-mouse, 1:1000; Vector Laboratories) or FITC (goat anti-rabbit, 1:1000; Vector Laboratories) were applied for 45 min at 37°C in 3% BSA. Finally, cells were mounted with Mowiol 4-88 (Carl Roth) and viewed under a fluorescence microscope (Zeiss). To confirm the specificity of staining all procedures were also performed without the first antibody.

### Western blot

The dissected tissue was mechanically solubilised in lysis buffer containing (mM): 50 Tris (pH 7.4), 5 EDTA, 150 NaCl, 0.5% NP40, 0.5% Na desoxycholat, incubated for 30 min and centrifuged for 20 min at 13,000 rpm at 4°C. The protein concentration was quantified using the Bradford protein assay (Biorad). 50–75 µg of protein was separated by SDS–PAGE and transferred onto a nitrocellulose membrane (Nylon N^+^ membrane, Amersham). The membrane was incubated overnight at 4°C in primary anti-GIRK1 or anti-GIRK2 diluted 1:200 in blocking solution containing 5% milk powder (Carl Roth) in TBS-T. After incubation with an anti-rabbit peroxidase-conjugated secondary antibody (1:3000 in TBS-T; Jackson Immuno Research) the protein bands were detected by enhanced chemiluminescence (ECL; GE-Healthcare) according to the manufacturer's instructions.

### Generation of Na_v_1.8-GIRK2 mice

A mouse BAC clone (RP23-391G10) encompassing a 134 kb promoter region of s*cn10a* (coding for Na_v_1.8) and another complete gene s*cn11a* (coding for Na_v_1.9) was modified in several steps. This led to a BAC encompassing a 113 kb promoter region of *scn10a* followed by the sequence of the flag-GIRK2-polyA. The genes of Na_v_1.8 and Na_v_1.9 were trimmed in order to avoid functional expression of the two genes. For transgenic mouse generation the modified BAC construct was injected into fertilised oocytes, which were transplanted into the oviduct of foster mothers.

For quantitative analysis of transgene copy number, Southern blot and real-time PCR was used. Southern blot copy number standards were established with 10 µg of genomic wildtype DNA isolated from tail biopsies and spiked with 1, 2, 4, 8 and 16 BAC copies. Copy number was determined by comparing pixel intensity of the band of Na_v_1.8-GIRK2 mice with the standards. For qRT-PCR a standard curve was calculated based on genomic wildtype DNA spiked with 2, 4, 6, 8, 16 and 32 BAC copies. Copy number of Flag-GIRK2 in Na_v_1.8-GIRK2 mice was compared to the standards.

### K^+^-Imaging

Dissociated DRG neurons were used 12–24 h after plating and data were analyzed using Tillvision software (Till Photonics). The FluxOR™ Potassium Ion Channel Assay (Invitrogen) was performed according to the manufacturer's instructions. Briefly, DRG neurons were incubated for 90 min with 1 ml of the loading buffer containing the thallium sensitive dye. Neurons were washed three times with PBS following a pre-incubation for 30 min with assay buffer containing Tetraethylammonium chloride (20 mM) and Glybenclamide (20 µM) to block other K^+^ channels. Fluorescence was measured at excitation wavelength of 495 nm for 5 min. Cells were treated with DAMGO (10 µM) or DPDPE (10 µM) diluted in stimulus buffer containing the Tl_2_SO_4_ or with stimulus buffer only for control. The fluorescence of each cell was normalised to baseline. For statistical analysis the area under the curve (AUC) was calculated.

### Animals

All animal experiments were approved by the local animal committee (Landesamt für Gesundheit und Soziales Berlin, Germany). Adult male Na_v_1.8-GIRK2 mice, adult male wildtype C57Bl6-J littermates (bred at Charité-Universitätsmedizin Berlin, Campus Benjamin Franklin) or adult male Wistar rats (Janvier, France) were used for behaviour experiments.

## Nociceptive testing

Thermal hyperalgesia was analyzed using the Hargreaves test. Radiant heat was applied to the plantar surface of a hind paw with a high-intensity light beam, and paw withdrawal latency was measured with an electronic timer (IITC Inc/Life Science, Woodland Hills, CA). The cut-off was 20 s to avoid tissue damage. Measurements were done at least twice (mice) or three times (rats) on each paw and the means were used for statistical analysis.

Mechanical allodynia in mice was evaluated as described elsewhere (Labuz et al, 2009) using calibrated von Frey filaments (Stoelting, Wood Dale, IL). In rats the plantar surface of each hindpaw was stimulated with von Frey filaments (Stoelting, Wood Dale, IL, USA) of increasing force until the filament, which gave withdrawal responses to three stimuli was reached. The strength of calibrated von Frey filaments was 0.56, 0.96, 1.27, 2.12, 3.77, 6.05, 7.50, 9.94, 14.32, 27.67 g.

## Induction of inflammation and neuropathy

Twenty microliters of Complete Freund's adjuvant (CFA; 50 µg of Mycobacterium Butyricum dissolved in 20 µl incomplete Freund's adjuvant; DIFCO) were injected with a 28G needle into mouse right hind paws. Rat paws were injected with 150 µl CFA. The injection was performed under light isoflurane anaesthesia. Thermal and mechanical thresholds were measured before, 2 h (data not shown) and 2 days after injection as described above. Neuropathy was induced by chronic constriction injury (CCI) of the sciatic nerve as described elsewhere (Labuz et al, 2009). Thermal and mechanical thresholds were measured before, 2 and 14 days after CCI as described above (data not shown).

## Pharmacology

The antinociceptive effect of DAMGO (0.05–64 µg) injected intraplantarly (i.pl.) into inflamed hindpaws of mice (20 µl) or rats (100 µl) under brief isoflurane anaesthesia was determined as described above. Control groups were treated with 0.9% NaCl. The effect of i.pl. DAMGO in mice and rats was assessed 5 min after drug application. The time point and concentration of maximum effect were used to test for opioid receptor specificity with the unselective opioid receptor antagonist naloxone-methiodide (NLXM; 5 µg) injected together with DAMGO into the paw. This antagonist is not able to cross the blood–brain barrier, thus it acts only in the periphery. The experimenter was blinded to the treatments and the genotype of the animals.

## Analysis of *Kcnj3* and *Kcnj6* promoters

GIRK1 genomic sequence is well annotated in mouse, human and rat databases while GIRK2 sequence is annotated only in mouse and human. Information on GIRK2 is incomplete in ENSEMBL and UCSC databases. We therefore used the Celera genomic dataset and EST trace libraries to assemble a rat genomic annotated region as described in Supplementary Methods. To functionally analyze differences between mouse and rat promoters (insertions and deletions larger than 30 bases), fragments of the promoters were subcloned into a promoter-less eGFP expression reporter using PCR (see Supplementary Methods for details). Reporter constructs were transfected into DRG neurons using the Amaxa Nucleofector II System with the Basic Neuron SCN Nucleofector Kit following the manufacturer's protocol.

## Data Analysis and statistics

All data are presented as mean ± SEM For data analysis and plot drawing SigmaStat and SigmaPlot (Systat) software was used. Two-sample comparisons were made with the Student's *t*-test, or, for non-parametric data, with the Mann–Whitney rank sum test or, for repeated measurements, with the Wilcoxon Signed Rank test. For more than two experimental groups a one-way ANOVA was used. A Kruskal–Wallis ANOVA on Ranks was used for non-parametric data. If two different factors in more than two groups were verified a two-way ANOVA was used. Both were followed by a Holm–Sidak test for comparison versus a control group. For repeated measurements a one-way or two-way repeated ANOVA was used, followed by Bonferroni test for pairwise comparison or by Holm–Sidak test if the data were compared to a control group.

The use of opioid agonists acting outside the CNS is a promising therapeutic strategy for pain control. However, the efficacy of peripherally applied opioids varies across species. For example, in human and rats peripherally restricted opioids show powerful analgesic effects, while in mice their action remains unclear.

RESULTS:

We found that GIRK K^+^ channels, major effectors for opioid signalling in the CNS, are absent from mouse peripheral sensory neurons but present in human and rat. *In vivo* transgenic expression of GIRK channels in mouse nociceptors established peripheral opioid signalling and local analgesia. We further identified a regulatory element in the rat GIRK2 gene that accounts for differential expression in rodents.

IMPACT:

Our data indicate that GIRK channels are indispensable for peripheral opioid analgesia, and their expression in rodent sensory neurons underlies the differential sensitivity of peripherally applied opioids in rats and mice. The absence of GIRK channels from mouse DRG, raises concerns over the predictive validity of mice as a model organism for investigating peripheral GPCR-mediated analgesia.

## Author contributions

DN, CS and PAH designed experiments. DN, DL, MR, FCR and KMK performed experiments. PH performed computational analysis and designed experiments for promoter studies. DN, CS and PAH prepared the manuscript.

## Supporting Information

As a service to our authors and readers, this journal provides supporting information supplied by the authors. Such materials are peer reviewed and may be re-organised for online delivery, but are not copy-edited or typeset. Technical support issues arising from supporting information (other than missing files) should be addressed to the authors.

**Figure S1. Ion channel modulation by opioid receptors.** (**A** and **B**) Representative whole cell calcium channel current traces of dissociated mouse DRG neurons evoked by voltage steps from a holding potential of −90 mV to +10 mV. (A) Calcium channel currents were reduced in the presence of the µ-opioid receptor agonist DAMGO (10 µM; (**p* = 0.031; Wilcoxon Signed Rank Test; *n* = 6)). (B) Naloxone (20 µM) reversed the inhibition of calcium channel currents produced by DAMGO (*p* = 0.625; Wilcoxon Signed Rank Test; *n* = 5). (**C**) Example of the current–voltage relationship in a HEK293 cell transfected with GIRK1, GIRK2 and the µ-opioid receptor. Expression of GIRK channels produced large inward currents upon hyperpolarization of the membrane, which were increased by DAMGO (10 µM) and inhibited by tertiapin Q (100 nM). (**D**) Quantification of peak inward currents at −120 mV (**p* = 0.002; one-way repeated measures ANOVA on ranks on raw values, *n* = 4–7). (**E**) Example of the current–voltage relationship in a control mouse DRG neuron and in a mouse DRG neuron infected with an adeno-associated viral vector-GIRK2 construct (AAV-GIRK2). (**F**) Immunoreactivity for δ opioid receptor in normal mouse DRG sections. (**G**) Immunoblot analysis of GIRK channels in mouse DRG neurons and spinal cord. Tissue lysates were stained with anti-GIRK1 and anti-GIRK2. Fifty micrograms of total protein was loaded per lane. (H) Representative current traces of Nav1.8-GIRK2 DRG neurons voltageclamped at −80 mV showing no major desensitization after repeated application of 10 µM DAMGO. **Figure S2. Generation and characterization of Nav1.8-GIRK2 mice.** (**A**) Nine copies of the transgene integrated into the genome of Nav1.8-GIRK2 mice, as determined by Southern blot of tail DNA from Nav1.8-GIRK2 mice (upper panel) and qRT-PCR analysis (lower panel). (**B**) Analysis of GIRK2 mRNA expression in DRG neurons before and 48 h after induction of unilateral hindpaw inflammation or chronic constriction injury of the sciatic nerve. (**C**) Dissociated DRG neurons isolated from Nav1.8-GIRK2 mice immunostained with anti-FLAG and co-stained with anti-GIRK2, anti-CGRP, IB4 or anti-µ opioid receptor. (**D** and **E**) DAMGO-induced thallium uptake in dissociated DRG neurons loaded with the thallium-sensitive dye FluxOR. (D) Time course of thallium uptake in Nav1.8-GIRK2 and wildtype DRG neurons measured as fluorescence emission at 525 nm, normalized to baseline. (E) Integrated fluorescence measured in wildtype and Nav1.8-GIRK2 DRG neurons treated with 10 µM DAMGO. Control displays the fluorescence increase in DRG neurons without stimulation with opioid receptor agonists (****p* < 0.001; Mann–Whitney Rank Sum test, *n* = 200–300 cells per group from four animals). (**F**) Paw withdrawal latency of naïve Nav1.8-GIRK2 and wildtype mice measured at different time points after injection of DAMGO or NaCl into the right hind paw (i.pl.). No differences were detected at baseline or after the injection of DAMGO (*p* > 0.05; two-way repeated ANOVA, *n* = 8). All scale bars are 50 µm. **Figure S3. Antinociceptive effect of peripherally applied DAMGO in male Wistar rats.** (**A**) Changes in paw withdrawal latency in response to radiant heat upon induction of inflammation (ipsilateral) compared to the noninflamed (contralateral) side. (**B**) Dose-dependent inhibition of thermal hyperalgesia 5 min after DAMGO injection into the inflamed paw (****p* < 0.001, **p* = 0.031; one-way ANOVA, Bonferroni *t*-test, *n* = 6–8). (**C**) DAMGO-induced antinociception is reversed by co-injection of naloxone-methiodide (NLXM, 5 µg) in the inflamed paw (***p* = 0.007; Student's unpaired *t*-test, *n* = 8). (**D**) Changes in paw withdrawal threshold in response to mechanical stimuli in the inflamed (ipsilateral) and noninflamed (contralateral) paws of rats. (**E**) Dose-dependent inhibition of mechanical allodynia 5 min after DAMGO injection into the inflamed paw (****p* < 0.001, ***p* = 0.002; one-way ANOVA, Bonferroni *t*-test, *n* = 8). (**F**) DAMGO-induced antinociception is reversed by co-injection of NLXM (5 µg) in the inflamed paw (***p* = 0.002; Student's unpaired *t*-test, *n* = 8). **Figure S4.** A**bsence of eGFP expression in DRG neurons transfected with mouse and shorter rat reporter constructs.** (**A**) Mouse reporters proximal to position 1052 relative to the transcription start-site of *Kcnj6* do not drive eGFP expression in mouse sensory neurons. Top images indicate the lack of fluorescence for the equivalent phase contrast image below. (**B**) Rat reporter constructs shorter than 1043 nucleotides from the *Kcnj6* transcription start-site do not induce eGFP fluorescence in transfected mouse sensory neurons. All scale bars are 100 mm. **Figure S5. Absence of eGFP expression in cerebellum cultures transfected with mouse and rat reporter constructs.** (**A**) Transfection of CMV-eGFP in neonatal mouse cerebellum cultures induces strong fluorescence indicating efficient transfection. Mouse reporters proximal to position 1052 relative to the transcription start-site of *Kcnj6* do not drive eGFP expression in cerebellum neurons. Top images indicate the lack of fluorescence for the equivalent phase contrast image below. (**B**) Rat reporter constructs shorter than 1195 nucleotides from the *Kcnj6* transcription start-site do not induce eGFP fluorescence in transfected cerebellum neurons. All scale bars are 100 mm. **Figure S6. Alignment of rat and mouse strain genomic sequence upstream of the *Kcnj6* transcription start site.**.
