# Supplementary material for: The K+ channel GIRK2 is both necessary and sufficient for peripheral opioid-mediated analgesia
Source: EMBO Mol Med. 2013 Jul 1;5(8):1263–77. doi: 10.1002/emmm.201201980 (PMC3944465; doi:10.1002/emmm.201201980)
Supplement: Supplementary file 1 — Figure S1. Ion channel modulation by opioid receptors. (A and B) Representative whole cell calcium channel current traces of dissociated mouse DRG neurons evoked by voltage steps from a holding potential of −90 mV to +10 mV. (A) Calcium channel currents were reduced in the presence of the µ-opioid receptor agonist DAMGO (10 µM; (*p = 0.031; Wilcoxon Signed Rank Test; n = 6)). (B) Naloxone (20 µM) reversed the inhibition of calcium channel currents produced by DAMGO (p = 0.625; Wilcoxon Signed Rank Test; n = 5). (C) Example of the current–voltage relationship in a HEK293 cell transfected with GIRK1, GIRK2 and the µ-opioid receptor. Expression of GIRK channels produced large inward currents upon hyperpolarization of the membrane, which were increased by DAMGO (10 µM) and inhibited by tertiapin Q (100 nM). (D) Quantification of peak inward currents at −120 mV (*p = 0.002; one-way repeated measures ANOVA on ranks on raw values, n = 4–7). (E) Example of the current–voltage relationship in a control mouse DRG neuron and in a mouse DRG neuron infected with an adeno-associated viral vector-GIRK2 construct (AAV-GIRK2). (F) Immunoreactivity for δ opioid receptor in normal mouse DRG sections. (G) Immunoblot analysis of GIRK channels in mouse DRG neurons and spinal cord. Tissue lysates were stained with anti-GIRK1 and anti-GIRK2. Fifty micrograms of total protein was loaded per lane. (H) Representative current traces of Nav1.8-GIRK2 DRG neurons voltageclamped at −80 mV showing no major desensitization after repeated application of 10 µM DAMGO. Figure S2. Generation and characterization of Nav1.8-GIRK2 mice. (A) Nine copies of the transgene integrated into the genome of Nav1.8-GIRK2 mice, as determined by Southern blot of tail DNA from Nav1.8-GIRK2 mice (upper panel) and qRT-PCR analysis (lower panel). (B) Analysis of GIRK2 mRNA expression in DRG neurons before and 48 h after induction of unilateral hindpaw inflammation or chronic constriction injury of the sciatic nerve. (C) Di [file emmm0005-1263-SD1.pdf]

Supplementary Information

Table of Contents

Supplementary Figure 1	Page 2
Supplementary Figure 2	Page 4
Supplementary Figure 3	Page 6
Supplementary Figure 4	Page 8
Supplementary Figure 5	Page 9
Supplementary Figure 6	Page 10
Supplementary Methods	Page 11
Supplementary Table	Page 12

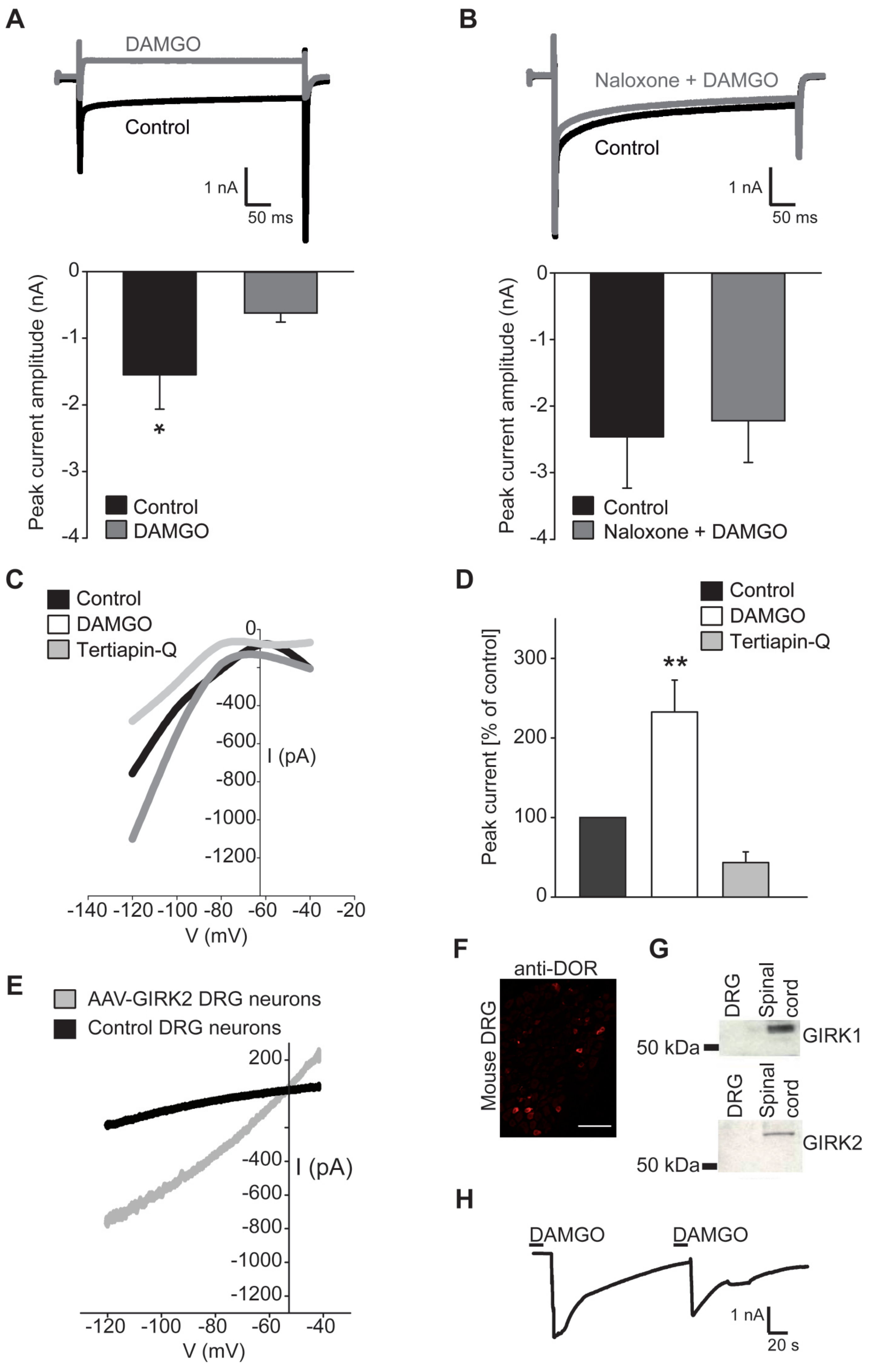
A
DAMGO
Control
1 nA
50 ms
Peak current amplitude (nA)
0
-1
-2
-3
-4
*
Control
DAMGO
B
Naloxone + DAMGO
Control
1 nA
50 ms
Peak current amplitude (nA)
0
-1
-2
-3
-4
Control
Naloxone + DAMGO
C
Control
DAMGO
Tertiapin-Q
0
-400
-600
-800
-1000
-1200
I (pA)
-140 -120 -100 -80 -60 -40 -20
V (mV)
D
Control
DAMGO
Tertiapin-Q
Peak current [% of control]
300
200
100
0
**
E
AAV-GIRK2 DRG neurons
Control DRG neurons
200
-400
-600
-800
-1000
-1200
I (pA)
-120 -100 -80 -60 -40
V (mV)
F
anti-DOR
Mouse DRG
G
DRG
Spinal cord
GIRK1
50 kDa
DRG
Spinal cord
GIRK2
50 kDa
H
DAMGO
DAMGO
1 nA
20 s

Supplementary Figure 1. Ion channel modulation by opioid receptors. (a and b) Representative whole cell calcium channel current traces of dissociated mouse DRG neurons evoked by voltage steps from a holding potential of -90 mV to + 10 mV. (a) Calcium channel currents were reduced in the presence of the μ-opioid receptor agonist DAMGO (10 μM; (*p = 0.031; Wilcoxon Signed Rank Test; n = 6)). (b) Naloxone (20 μM) reversed the inhibition of calcium channel currents produced by DAMGO (p = 0.625; Wilcoxon Signed Rank Test; n = 5). (c) Example of the current–voltage relationship in a HEK293 cell transfected with GIRK1, GIRK2 and the μ-opioid receptor. Expression of GIRK channels produced large inward currents upon hyperpolarization of the membrane which were increased by DAMGO (10 μM) and inhibited by tertiapin Q (100 nM). (d) Quantification of peak inward currents at -120 mV (*p = 0.002; one way repeated measures ANOVA on ranks on raw values, n = 4-7). (e) Example of the current-voltage relationship in a control mouse DRG neuron and in a mouse DRG neuron infected with an adeno-associated viral vector-GIRK2 construct (AAV-GIRK2). (f) Immunoreactivity for δ opioid receptor in normal mouse DRG sections. (g) Immunoblot analysis of GIRK channels in mouse DRG neurons and spinal cord. Tissue lysates were stained with anti-GIRK1 and anti-GIRK2. 50 μg of total protein was loaded per lane. (h) Representative current traces of Nav1.8-GIRK2 DRG neurons voltage-clamped at -80 mV showing no major desensitization after repeated application of 10 μM DAMGO.

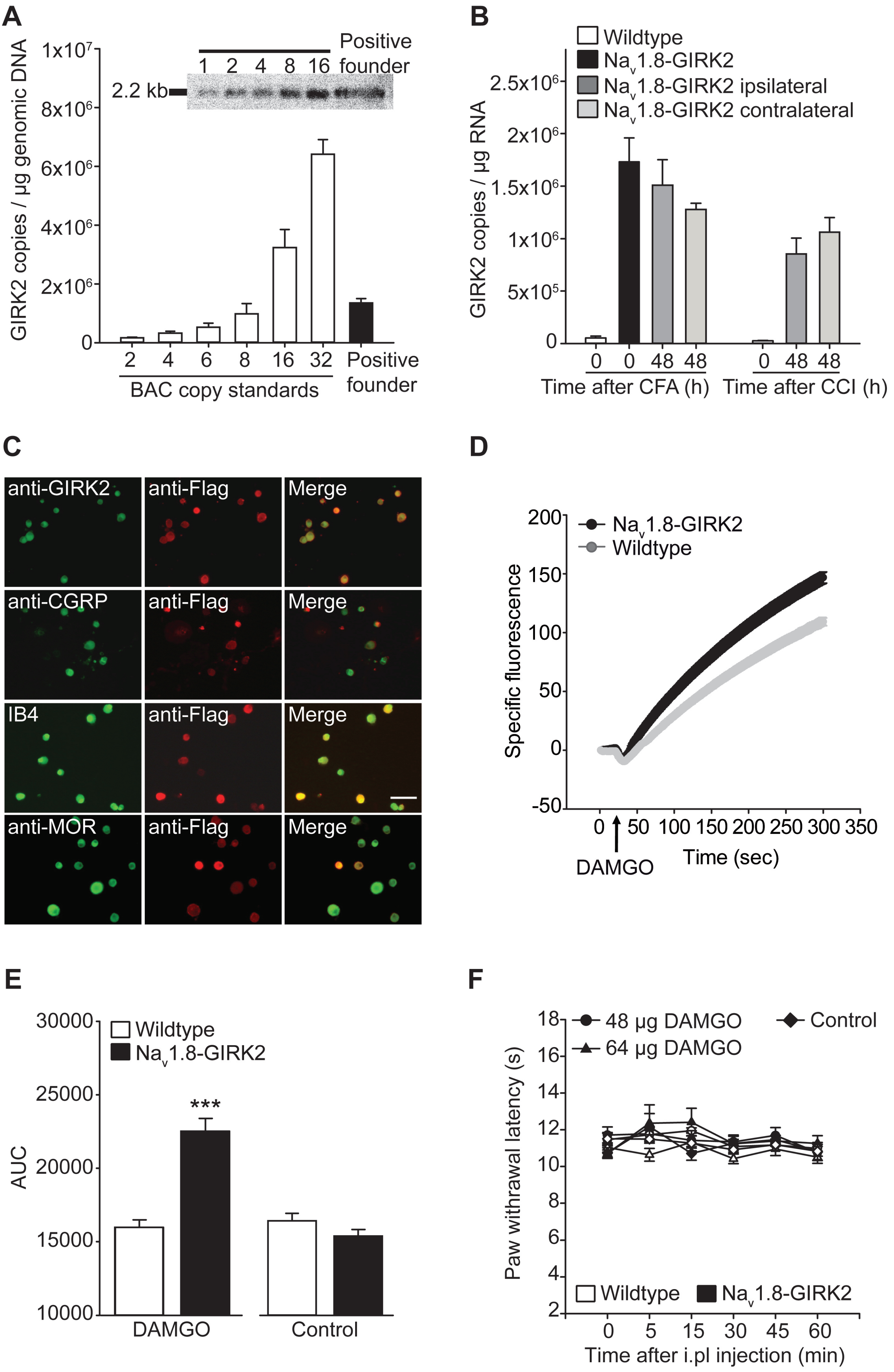
A
GIRK2 copies / µg genomic DNA
1x10^7
8x10^6
6x10^6
4x10^6
2x10^6
0
Positive founder
1 2 4 8 16
2.2 kb
2 4 6 8 16 32
BAC copy standards
Positive founder
B
Wildtype
Nav1.8-GIRK2
Nav1.8-GIRK2 ipsilateral
Nav1.8-GIRK2 contralateral
GIRK2 copies / µg RNA
2.5x10^6
2x10^6
1.5x10^6
1x10^6
5x10^5
0
0 0 48 48
Time after CFA (h)
0 48 48
Time after CCI (h)
C
anti-GIRK2
anti-Flag
Merge
anti-CGRP
anti-Flag
Merge
IB4
anti-Flag
Merge
anti-MOR
anti-Flag
Merge
D
Nav1.8-GIRK2
Wildtype
Specific fluorescence
200
150
100
50
0
-50
0 50 100 150 200 250 300 350
DAMGO
Time (sec)
E
Wildtype
Nav1.8-GIRK2
AUC
30000
25000
20000
15000
10000
***
DAMGO
Control
F
48 µg DAMGO
64 µg DAMGO
Control
Paw withrawal latency (s)
18
16
14
12
10
8
6
4
2
Wildtype
Nav1.8-GIRK2
0 5 15 30 45 60
Time after i.pl injection (min)

Supplementary Figure 2. Generation and characterization of Nav1.8-GIRK2 mice. (a) Nine copies of the transgene integrated into the genome of Nav1.8-GIRK2 mice, as determined by Southern blot of tail DNA from Nav1.8-GIRK2 mice (upper panel) and qRT-PCR analysis (lower panel). (b) Analysis of GIRK2 mRNA expression in DRG neurons before and 48 h after induction of unilateral hindpaw inflammation or chronic constriction injury of the sciatic nerve. (c) Dissociated DRG neurons isolated from Nav1.8-GIRK2 mice immunostained with anti-FLAG and co-stained with anti-GIRK2, anti-CGRP, IB4 or anti-μ opioid receptor. (d and e) DAMGO-induced thallium uptake in dissociated DRG neurons loaded with the thallium-sensitive dye FluxOR. (d) Time course of thallium uptake in Nav1.8-GIRK2 and wildtype DRG neurons measured as fluorescence emission at 525 nm, normalized to baseline. (e) Integrated fluorescence measured in wildtype and Nav1.8-GIRK2 DRG neurons treated with 10 μM DAMGO. Control displays the fluorescence increase in DRG neurons without stimulation with opioid receptor agonists (*** $p < 0.001$; Mann-Whitney Rank Sum test, $n = 200$-300 cells per group from 4 animals). (f) Paw withdrawal latency of naïve Nav1.8-GIRK2 and wildtype mice measured at different time points after injection of DAMGO or NaCl into the right hind paw (i.pl.). No differences were detected at baseline or after the injection of DAMGO ($P>0.05$; two-way repeated ANOVA, $n = 8$). All scale bars are 50 μm.

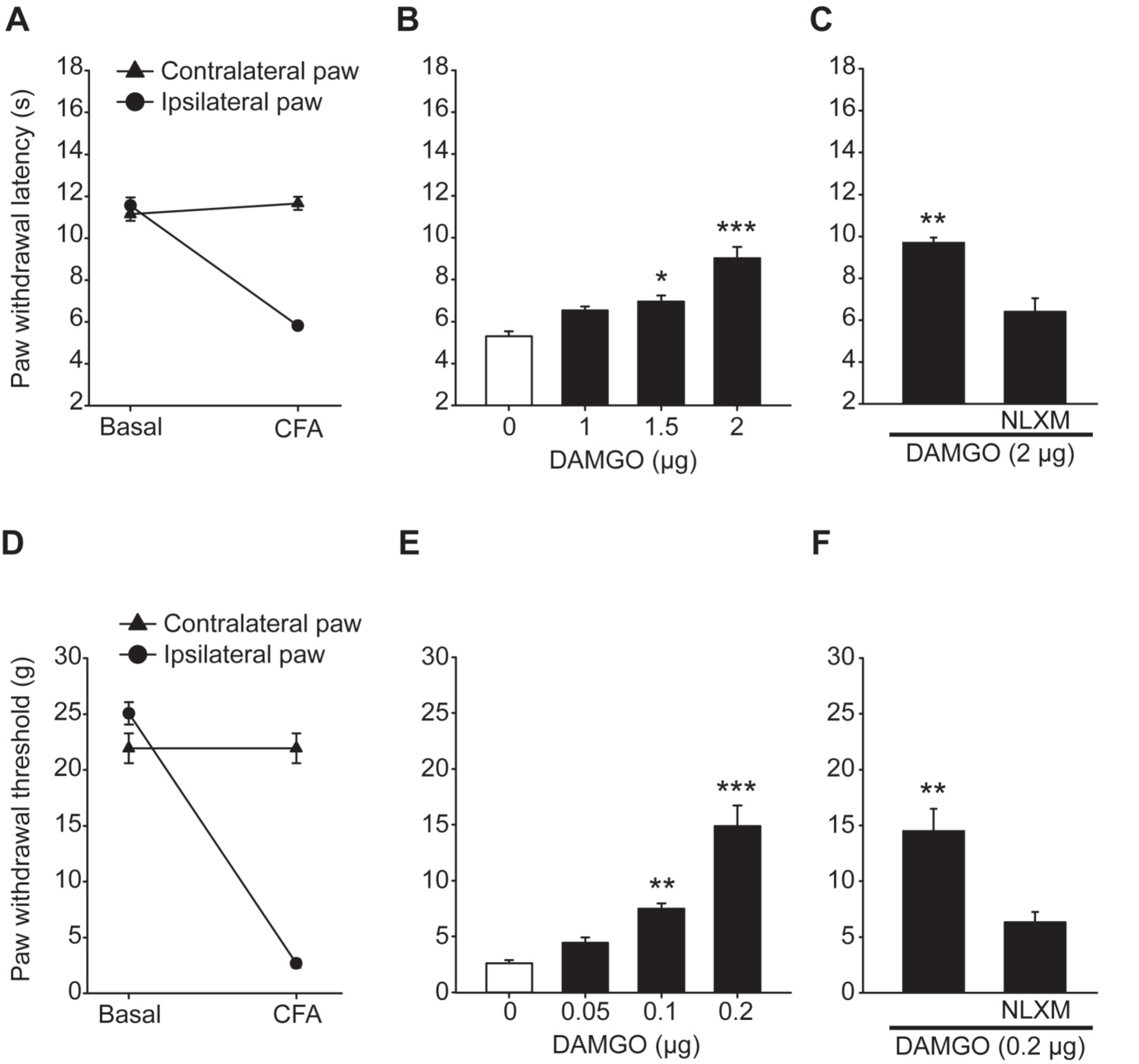
A
B
C
Paw withdrawal latency (s)
Contralateral paw
Ipsilateral paw
Basal
CFA
DAMGO (μg)
NLXM
DAMGO (2 μg)
D
E
F
Paw withdrawal threshold (g)
DAMGO (μg)
DAMGO (0.2 μg)

Supplementary Figure 3. Antinociceptive effect of peripherally applied DAMGO in male Wistar rats. (a) Changes in paw withdrawal latency in response to radiant heat upon induction of inflammation (ipsilateral) compared to the noninflamed (contralateral) side. (b) Dose-dependent inhibition of thermal hyperalgesia 5 min after DAMGO injection into the inflamed paw (***$p < 0.001$, *$p = 0.031$; one way ANOVA, Bonferroni t-test, $n = 6$-8). (c) DAMGO-induced antinociception is reversed by co-injection of naloxone-methiodide (NLXM, 5µg) in the inflamed paw (** $p = 0.007$; student´s unpaired t-test, $n = 8$). (d) Changes in paw withdrawal threshold in response to mechanical stimuli in the inflamed (ipsilateral) and noninflamed (contralateral) paws of rats. (e) Dose-dependent inhibition of mechanical allodynia 5 min after DAMGO injection into the inflamed paw (*** $p < 0.001$, ** $p = 0.002$; one way ANOVA, Bonferroni t-test, $n = 8$). (f) DAMGO-induced antinociception is reversed by co-injection of NLXM (5µg) in the inflamed paw (**$p = 0.002$; student´s unpaired t-test, $n = 8$).

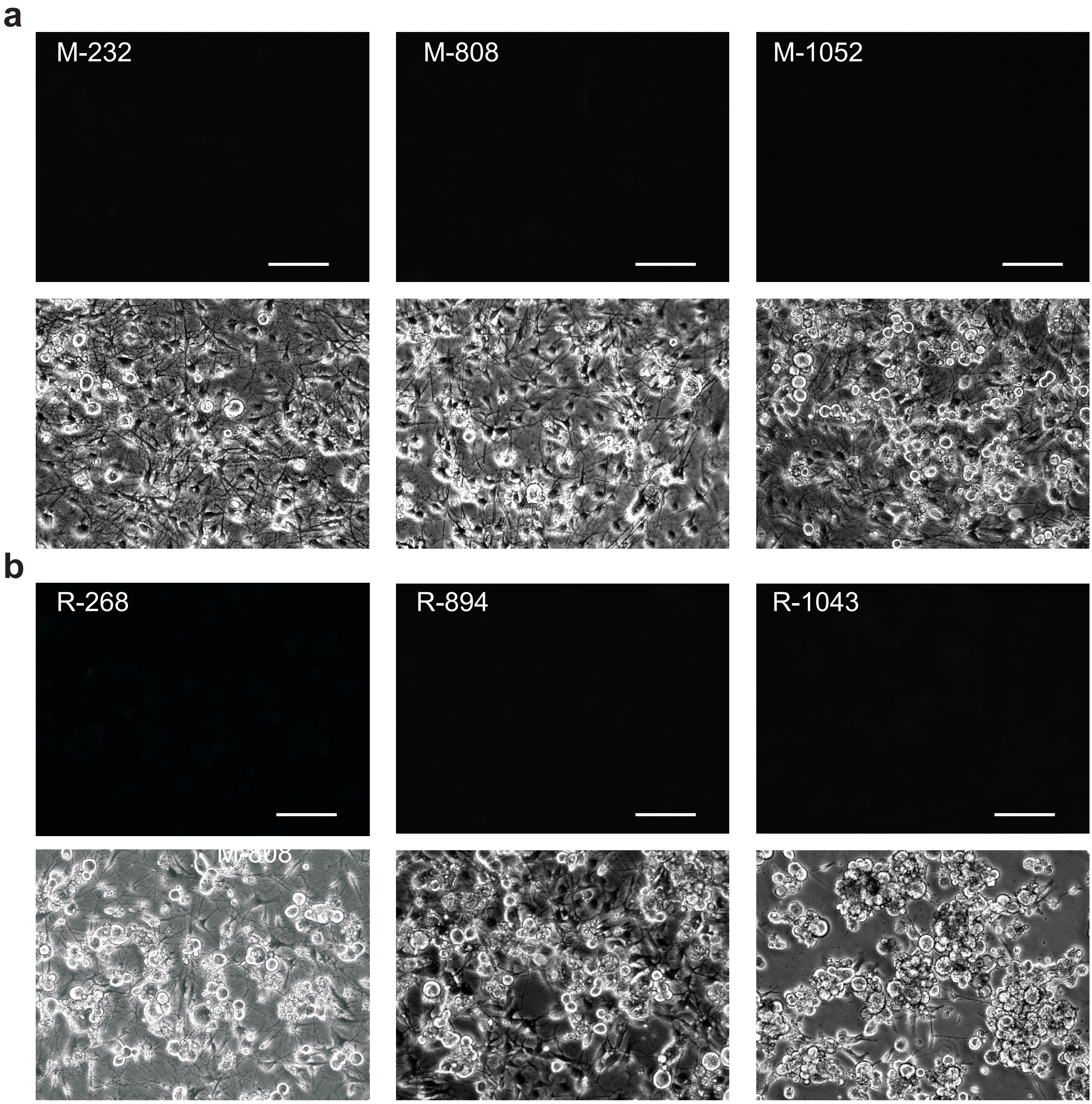

Supplementary Figure 4. Absence of eGFP expression in DRG neurons transfected with mouse and shorter rat reporter constructs. (**a**) Mouse reporters proximal to position 1052 relative to the transcription start-site of *Kcnj6* do not drive eGFP expression in mouse sensory neurons. Top images indicate the lack of fluorescence for the equivalent phase contrast image below. (**b**) Rat reporter constructs shorter than 1043 nucleotides from the *Kcnj6* transcription start-site do not induce eGFP fluorescence in transfected mouse sensory neurons. All scale bars are 100mm.

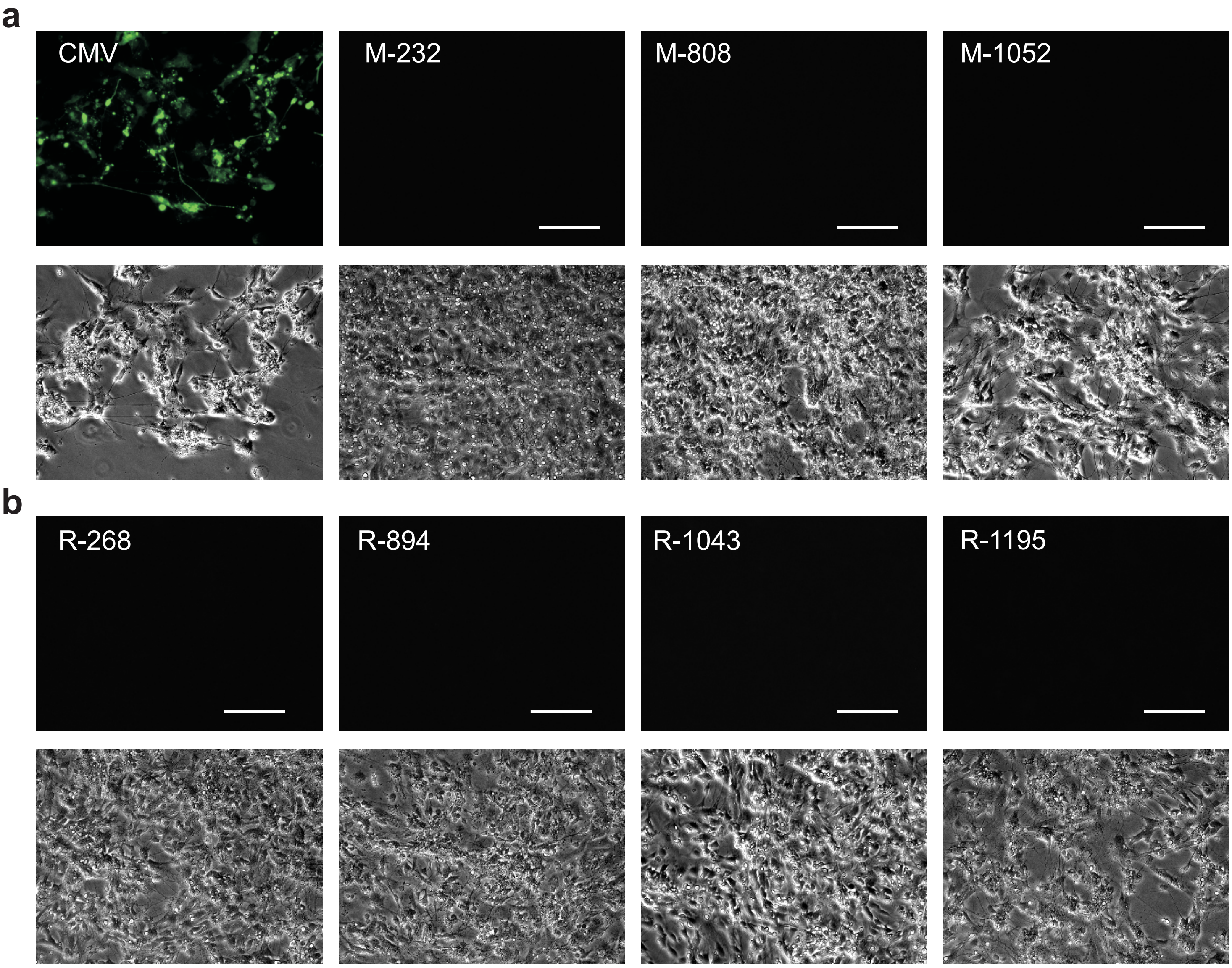

Supplementary Figure 5. Absence of eGFP expression in cerebellum cultures transfected with mouse and rat reporter constructs. (**a**) Transfection of CMV-eGFP in neonatal mouse cerebellum cultures induces strong fluorescence indicating efficient transfection. Mouse reporters proximal to position 1052 relative to the transcription start-site of *Kcnj6* do not drive eGFP expression in cerebellum neurons. Top images indicate the lack of fluorescence for the equivalent phase contrast image below. (**b**) Rat reporter constructs shorter than 1195 nucleotides from the *Kcnj6* transcription start-site do not induce eGFP fluorescence in transfected cerebellum neurons. All scale bars are 100mm.

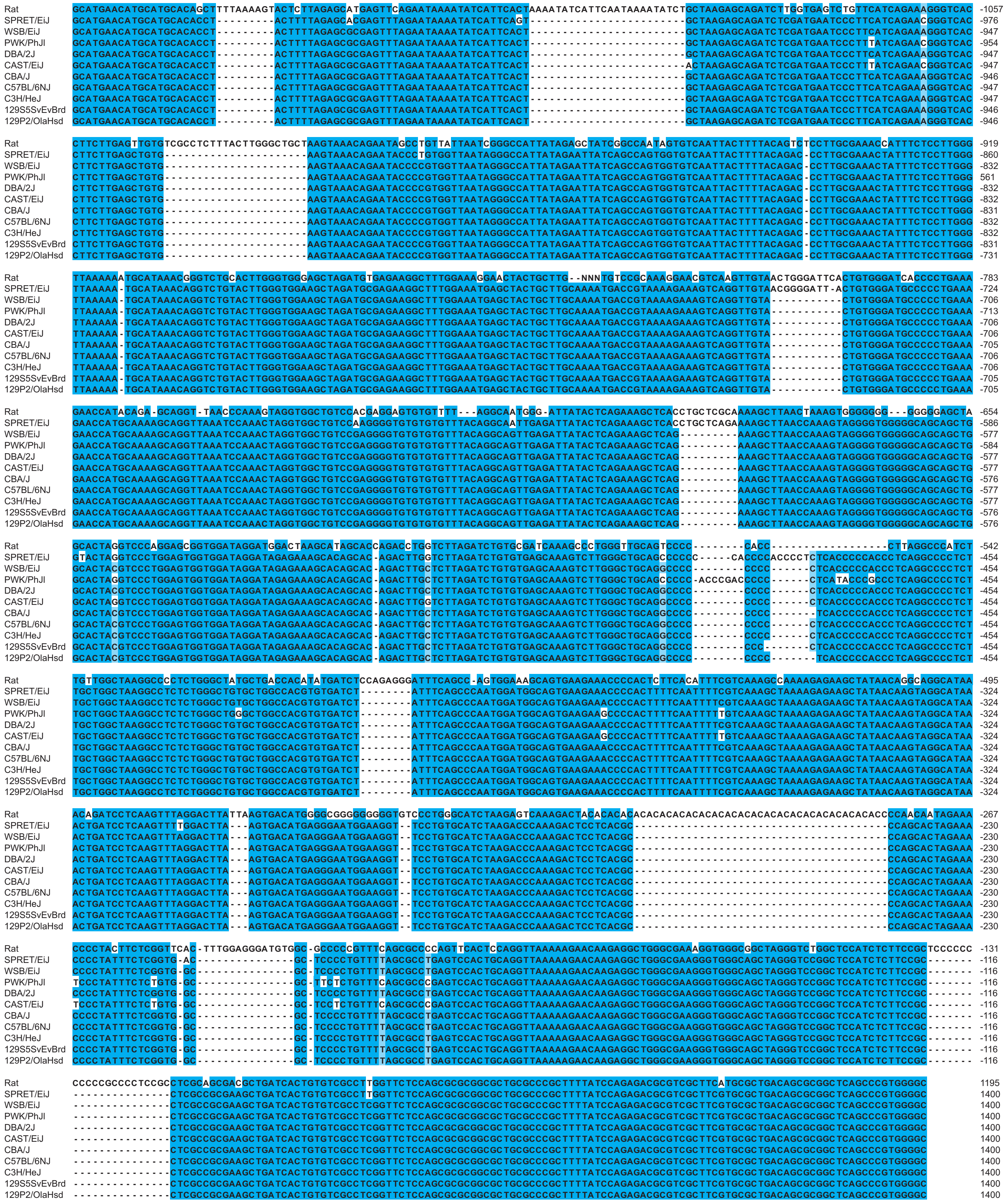

Supplementary Figure 6. Alignment of rat and mouse strain genomic sequence upstream of the *Kcnj6* transcription start site.

SUPPLEMENTARY METHODS

Primer sequences

GIRK1 and GIRK2 specific primers for qRT-PCR were selected as follows:

GIRK1-mouse/rat-forward 5´- ACCTGAACAAAGCCCATGTC-3´

GIRK1-mouse/rat-reverse 5´-GTTGATCGGCCCCTGTACTA-3´

GIRK2-mouse-forward 5´- GATTCCATGGACCAGGATGT-3´

GIRK2-mouse-reverse 5´- CCATAACCGATGGTGGTTTC-3´

GIRK2-rat-forward 5´- TGCCTGATGTTCCGGGTAGGGG-3´

GIRK2-rat-reverse 5´-GCTCTGCCCGGTTAGCCAGC-3´

GIRK1-human-forward 5´- TCCGCGCAGATTCGCTGCAA-3

GIRK1-human-reverse 5´- CACACTGTAAGGTGGGGTGGGGA-3

GIRK2-human-forward 5´- AGCAGCCGCGAGCAGAATGG-3´

GIRK2-human-reverse 5´- AGGTCTCCCTCACGTTGCCGT-3

Mice were genotyped using Flag-GIRK2 specific primers:

Flag-GIRK2-forward 5´-TACAAAGACGATGACGACAA-3´,

Flag-GIRK2-reverse 5´-GGTGGTTTCTGTCTCTATGG-3´.

Analysis of the rat Kcnj6 promoter

The Celera genomic dataset and EST trace libraries allowed us to assemble a rat genomic annotated region in which all coding regions (exons 2 to 4 and also the 5’ UTR within exon 2) aligned well to both human and mouse. Identification of the rat promoter region and non-coding exon 1 was achieved by a low-complexity screen using orthologuous sequences, and the rat counterpart was identified as a similar-sized fragment with similar spacing to exon 2. The identified rat non-coding exon 1 (509 bases) has a 155bp and 299bp mismatch to mouse and human, respectively. Importantly, re-screening within 1,6 million bases in the rat genome did not produce significant hits, supporting the accurate identity of exon 1. Moreover, entity of the first exon is further corroborated by alignment of upstream elements (i.e. potential promoter region), in which all

species display high similarity, being almost 100% in the proximal promoter. Of note, the Celera database did not cover the entire rat genomic sequence, and subsequent cloning and sequencing revealed that the 483bp N insertion right after the proximal promoter indeed is a stretch of 604 bases that aligns very well to mouse and human genomic, further supporting the finding that indeed exon 1 and the promoter of rat Kcnj6 has been correctly identified.

Promote regions analyzed for Kcnj3 and Kcnj6 genes

Mouse *Kcnj3*	Human *KCNJ3*	Mouse *Kcnj6*	Rat *Kcnj6*	Human *KCNJ6*
-216	-198	-232	-268	-273
-612	-590	-808	-894	-672
-1253	-1222	-1052	-1043	-981
-1623	-1593		-1195	
-2165	-2190			

Numbers indicate the initial nucleotide position relative to the transcription start site for each reporter construct for Kcnj3 and Kcnj6 genes.
