# Supplementary material for: The K+ channel GIRK2 is both necessary and sufficient for peripheral opioid-mediated analgesia
Source: EMBO Mol Med. 2013 Jul 1;5(8):1263–77. doi: 10.1002/emmm.201201980 (PMC3944465; doi:10.1002/emmm.201201980)
Supplement: Supplementary file 2 [file emmm0005-1263-SD2.pdf]

EMBO
Molecular Medicine

The K+ channel GIRK2 is both necessary and sufficient for peripheral opioid-mediated analgesia

Dinah Nockemann, Morgane Rouault, Dominika Labuz, Philip Hublitz, Kate McKnelly Fernanda C. Reis, Christoph Stein & Paul A. Heppenstall

Corresponding authors: Paul Heppenstall, European Molecular Biology Laboratory (EMBL) and Christoph Stein, Charité Campus Benjamin Franklin

Review timeline:

Submission date:	05 September 2012
Editorial Decision:	15 November 2012
Revision received:	02 May 2013
Editorial Decision:	16 May 2013
Revision received:	24 May 2013
Accepted:	24 May 2013

Transaction Report:

(Note: With the exception of the correction of typographical or spelling errors that could be a source of ambiguity, letters and reports are not edited. The original formatting of letters and referee reports may not be reflected in this compilation.)

Editor: Roberto Buccione

1st Editorial Decision 15 November 2012

Thank you for the submission of your manuscript to EMBO Molecular Medicine.

We are very sorry for the delay in getting back to you with the Reviewers' evaluation on your work. Unfortunately, in this case we experienced unusual difficulties in securing three appropriate reviewers in a timely manner. Indeed, in consultation with my colleagues, we have decided to proceed based on two consistent evaluations.

You will see that while both Reviewers underline the considerable potential interest of your work, they also both raise some doubts on the model system and a number of other concerns that prevent us from considering publication at this time.

Reviewer 1 feels that the pain research methods used do not support robust in vivo confirmation and that the behavioural findings require better experimental support.

Reviewer 2 also considers that the conclusions are not fully supported by the experimental evidence. For instance, your finding that GIRK2 displays a preferential peripheral expression relies on insufficient experimental evidence.

While it is clear that publication of the paper cannot be considered at this stage, I am open to the submission of a substantially revised manuscript, provided, however, that the Reviewers' concerns

© EMBO

are fully addressed with additional experimental data where appropriate.

Please note that it is EMBO Molecular Medicine policy to allow a single round of revision only and that, therefore, acceptance or rejection of the manuscript will depend on the completeness of your responses included in the next, final version of the manuscript.

As you know, EMBO Molecular Medicine has a "scooping protection" policy, whereby similar findings that are published by others during review or revision are not a criterion for rejection. I understand that the amount of work that would be required to submit a revised version of your manuscript is significant, hence, should you decide to submit a revised version, I do ask you to get in touch with us after three months if you have not completed it, to update us on the status. Please also contact us as soon as possible if similar work is published elsewhere.

I look forward to seeing a revised form of your manuscript as soon as possible.

***** Reviewer's comments *****

Referee #2 (Comments on Novelty/Model System):

This manuscript claims new discoveries in GPCR signaling that impacts effectiveness of opioid analgesia in mice comparing to rats or humans. Behavioral pharmacology, molecular manipulations, in vitro electrophysiology, imaging and immunohistochemistry helped authors to demonstrate that the K+ channel GIRK2 is absent from mice peripheral sensory neurons comparing to rats and humans; convincing them to conclude that the lack of functional GIRK channels is responsible for the poor role of opioid mediating analgesia in all mice species.

As the paper is written, it is showing novelty and biological significance, but is weak showing behavioral evidence to the pain field. In other words, the paper clearly demonstrates that GIRK channels are absent in wild type mice (structural biology, in vitro functional electrophysiology), but the way pain research methods were used are not good enough to demonstrate robust in vivo confirmation (in vivo functional biology); making hard to see that the nonexistence of these channels fully accounts for the difference between opioid-mediated analgesia in mice versus opioid-mediated analgesia in rats or humans.

The behavioral findings are supported only by few experiments and incomplete description of the nociceptive techniques implemented. Behavioral studies compared baseline responses against two days after CFA injections for each mice group only (Fig. 4). It is well known that after a day of a single CFA injection the observed hyperalgesia response decays progressively. It would be better to repeat the assay and test the animals two hours after the CFA injection when they must show the greatest hyperalgesia seen in this model, or to substitute the CFA test with the formalin test. Authors used the CCI method to induce neuropathy in mice, but they do not show any behavioral data describing their results, only a follow up of two days checking for the GIRK2 mRNA expression in DRG neurons (Fig. S2). The full development of the mechanical and thermal hyperalgesia seen in the CCI model takes more than two days for both rats and mice. I suggest changing the CCI for SNI (spared nerve injury) model which has a better output in mice and to let the neuropathy to develop for at least 10 days. It would be a great value to the paper to divide and to describe behavior results in two groups: acute inflammatory pain and chronic neuropathic pain and once hyperalgesia is confirmed then proceed to test opioid mediated analgesia in both; also to add behavior data done in rats as an important standard point of comparison.

Referee #2 (General Remarks):

This manuscript claims new discoveries in GPCR signaling that impacts effectiveness of opioid analgesia in mice comparing to rats or humans. Behavioral pharmacology, molecular manipulations, in vitro electrophysiology, imaging and immunohistochemistry helped authors to demonstrate that the K+ channel GIRK2 is absent from mice peripheral sensory neurons comparing to rats and

humans; convincing them to conclude that the lack of functional GIRK channels is responsible for the poor role of opioid mediating analgesia in all mice species.

Major issues:

As the paper is written, it is showing novelty and biological significance, but is weak showing behavioral evidence to the pain field. In other words, the paper clearly demonstrates that GIRK channels are absent in wild type mice (structural biology, in vitro functional electrophysiology), but the way pain research methods were used are not good enough to demonstrate robust in vivo confirmation (in vivo functional biology); making hard to see that the nonexistence of these channels fully accounts for the difference between opioid-mediated analgesia in mice versus opioid-mediated analgesia in rats or humans.

The behavioral findings are supported only by few experiments and incomplete description of the nociceptive techniques implemented. Behavioral studies compared baseline responses against two days after CFA injections for each mice group only (Fig. 4). It is well known that after a day of a single CFA injection the observed hyperalgesia response decays progressively. It would be better to repeat the assay and test the animals two hours after the CFA injection when they must show the greatest hyperalgesia seen in this model, or to substitute the CFA test with the formalin test. Authors used the CCI method to induce neuropathy in mice, but they do not show any behavioral data describing their results, only a follow up of two days checking for the GIRK2 mRNA expression in DRG neurons (Fig. S2). The full development of the mechanical and thermal hyperalgesia seen in the CCI model takes more than two days for both rats and mice. I suggest changing the CCI for SNI (spared nerve injury) model which has a better output in mice and to let the neuropathy to develop for at least 10 days. It would be a great value to the paper to divide and to describe behavior results in two groups: acute inflammatory pain and chronic neuropathic pain and once hyperalgesia is confirmed then proceed to test opioid mediated analgesia in both; also to add behavior data done in rats as an important standard point of comparison.

Minor issues:

Subjects: mice strains used in this study are described in the results and not in the methods. Neither rat strain(s) used is/are described nor the origin of human samples used (e.g. where they came from, if the donors were healthy or not, etcetera).

Pain assessment: this subsection in the methods must be rewritten, as it is, is not solid. It would be better to describe first, all species used (sex, ages...); second, basal nociception tests, inflammatory test, neuropathic test; and third, the intraplantar pharmacology. Extra details in this subsection will be beneficial as the paper title lays on opioid-mediated analgesia with is directly linked to (in vivo) behavioral pharmacology.

Referee #3 (Comments on Novelty/Model System):

This is an elegant study that gives a molecular explanation to the modest peripheral analgesic effects of morphine in mice. However, the presented relationship to human is superficial. Deepning of this aspect would clearlly be of interest since the author started to address that. Technically, improvements should be provided.

Referee #3 (General Remarks):

The data presented by the authors provides a molecular explanation to the modest effects of peripheral opioids in mediating analgesia in mice. The authors first demonstrate that mouse and rat have major differences regarding this mechanism by showing that the GIRK channels are absent in mouse DRGs while present in rats. By creating a transgenic mouse model overexpressing GIRK2

channel specifically in sensory neurons mediating nociceptive detection, the author shows that peripheral opioid analgesia can be rescued in mouse. The study of the GIRK2 promoter region reveals that in the mouse, GIRK2 expression is silenced in DRGs by a unique deletion of a short regulatory element. The findings are totally novel and of interest to the community of physiologists and pharmacologists. The experiments are convincing although major complements should be added to be fully conclusive. Overall the manuscript is relatively clear and provides the reader with the background necessary for a good understanding of the results. I have anyway some question that would need to be addressed.

Specific points:

1)Fig1G: too dim signal to have an opinion. Please provide convincing example.

2)The reference to a "functional" expression of GIRK2 channels in human (in the text and in the figure2) is not supported at all. The only evidence provided is an example of labeling in fig2g. This is largely insufficient to claim that this is a functional expression in human DRGs. The author should be more convincing by showing more staining using flurorescence immunodetection as for mice sections. The expression in human DRG would be required also to claim that the channel is expressed in DRG... In addition the analysis done on the rat and mouse promoter region of GIRK2 should be also performed in human to conclude in the text that rat and human have a conserved mechanism.

3)The expression of the GIRK2-flag channels in the transgenic animals should be better described. It is hard to identify the neuronal DRG subtypes targeted by the transgene. A classical fluorescent immunodetection of the flag epitope together with nociceptive and non-nociceptive markers in DRG sections would be more appropriate (Flag + IB4, + CGRP, +NaV1.8, +peripherin, +NF200, etc...).

4)Since the transgene is not expressed in all DRG neurons, the authors should explain how they identify the neurons they are patching.

5)The authors provide evidence for a preferential peripheral expression of GIRK2 in the transgenic animals with a minimal if any targeting to the central synapse in the sensory neurons. Again the IHC data provided with DAB staining of the flag epitope are the only arguments. Other arguments should be provided such as dual immunodetection of the flag and the GIRK2 (anti flag and anti GIRK2 with 2 different fluorescent secondary). A western blot with an anti-flag on skin / sciatic nerve / DRG / and spinal cord should be presented. Ultimately, the use of the AAV-GIRK2 in vivo (injection in the sciatic nerve), would allow to see if the GIRK2 protein is restricted to the peripheral part of the DRGs or not.
Such absence of functional GIRK2 from the presynaptic sensory inputs in the spinal cord could be also probed by electrophysiological means in slices by comparing the DAMGO modulation of mini EPSP in WT and Tg animals.

6)The behavioral data shows that the analgesic doses of DAMGO are totally different in thermal and mechanical pain stimuli paradigms. This is rather spectacular and not discussed. An explanation should be provided. What kind of effect is achieved with 48µg on mechanical pain?

7)The method for transfecting DRG neurons in culture with the promoter-GFP plasmids is not explained.

Minor point:

1)Sup figure 1: please show tail current on the calcium channel traces as well as full time course of the experiment with DAMGO washout

2)Provide reference for the well-known expression of GIRK2 in keratinocytes. Staining in Girk2 KO?

1st Revision - authors' response 02 May 2013

Reviewer #1 (General Remarks):

This paper suffers from poorly presented electrophysiology data and this should be corrected before publication. Otherwise, the paper seems excellent and fully convinces me that the molecular pathway for peripheral opioid analgesia in sensory neurons is through G protein-coupled inward rectifier potassium channels (GIRK). The authors establish this so cleanly by taking advantage of a prior demonstration from the Stein lab that rats exhibit this analgesia whereas mice do not. The authors demonstrate that GIRK transfection in a transgenic mouse leads to peripheral opioid analgesia that is not found in wildtype mice. They further find an upstream regulatory element that appears to be the molecular basis for this difference between mice and rats. These results are highly significant for human health because peripheral opioid analgesia is effective and practical in humans.

Major Problems

Figure 1a. There are several problems with this data.

1) The legend says the currents were evoked with voltage ramps but the data are a series of discrete points at 20 mV intervals rather than the continuous record that occurs with a ramp.

In the original figure, discrete data points were extracted from ramp data. We have now corrected this and plotted the continuous ramp record.

2) The currents are described as inwardly rectifying, but they are not. They are more or less linear, with a slight upward bow and definitely not the strong downward bow of inward rectification.

We have modified the text and no longer refer to currents as inwardly rectifying.

3) It is neither standard nor reasonable to plot voltage with the more negative number to the right.

This has been corrected

Figure 1c. These currents are called representative but they are not. DAMGO induced current is much smaller than the average seen in Fig 1d (which is almost 2 nanoamps, a pretty big number) and current is not decreased by naloxone as is the average current.

We have now included representative currents.

Figure 1f. The absence of staining of DRG is uncompelling without some indication that the DRG can be labelled by some antibody; how do I know this is not a fixation artifact in this preparation?

Supplementary Figure 1F now shows a DRG section stained with an antibody against opioid receptors. In the same section and protocol, GIRK antibodies did not give a signal, implying that the lack of GIRK immunoreactivity is not due to a fixation or preparation artifact.

Figure 2a/b. In general, these data are inadequate because they fail to explore reproducibility of DAMGO currents in response to successive DAMGO applications, desensitization of the current, and inward rectification. The legend says that barium and naloxone decrease the DAMGO-induced current but this cannot be concluded because we have no idea how big this current should be. DAMGO current is clearly prolonged and decreases over time; this is reason to believe it desensitizes over time, which would invalidate comparisons of peak amplitudes of the first and second DAMGO applications. Thus, the data in 2b are invalid because they are plotted without consideration of the possibility that DAMGO-induced currents have desensitized by the time that the barium and naloxone data is gathered. In addition to failing to consider desensitization, there is no evidence given that these putative GIRK currents indeed exhibit inward rectification because currents are shown only for one voltage.

In these experiments we adhered to the following application protocol for each cell: DAMGO application followed by DAMGO plus naloxone, followed by washout, followed by DAMGO, followed by DAMGO plus barium. There was no significant difference between the first DAMGO application and the second (indeed these data points are pooled in Figure 2b), as illustrated in Supplementary Figure 1H. We are therefore confident that desensitization is not an issue here.

The Discussion is unnecessarily long. A specific problem is that it restates every result in the paper and takes several pages doing so. Please summarize results briefly and then discuss.

We have shortened the discussion.

The first sentence of the Abstract should not say "brain"; it should say "central nervous system". You must clearly distinguish analgesia in the dorsal horn of the spinal cord from peripheral analgesia studied in this paper, and "brain" does not do this. In fact, the Introduction could do a better job of saying that there are several places where analgesia is created: brain, spinal cord, dorsal root ganglion, and periphery.

This has been modified.

Minor comments

1) The term "Nav1.8-GIRK2" seems confusing to me. If I understand correctly, GIRK2 is being expressed via the driver for Nav1.8, but there is no change in Nav1.8 expression itself. If so, I would just call these GIRK2-expressing mice and explain in the text that Nav1.8 promoter is used to gain tissue specificity for sensory neurons. The term Nav1.8-GIRK suggests that both proteins are being altered.

Nav1.8-GIRK2 is the most conventional way of naming these mice (see for example HCN2 Ion Channels Play a Central Role in Inflammatory and Neuropathic Pain; Emery, et al.; Science 333, 1462 (2011)) and we would prefer to keep with this nomenclature. We have however clarified in the text the GIRK2 is being expressed from the Nav1.8 driver.

2) Figure 5 - the dotted blue line should be a colour other than blue. Either my eyes or the printer make it impossible to see the difference between the black and blue lines.

This has been changed

3) The references to Werz and Macdonald 1982 are a bit confusing because that work was based on action potentials, not currents. Distinguishing decreases in calcium flux from increases in potassium flux is not done cleanly in action potential measurements.

We have removed this reference.

4) Please state in the legend when high potassium solutions are used instead of normal saline to record GIRK currents.

This has been added.

5) On page 10, the sentence "Intriguingly, we were unable..." I suggest backing off this speculation. There have been many cases of speculations that a protein gets selectively transported to one end or the other of sensory neurons, and every one that has been explored carefully has been debunked. Very dangerous to speculate on a negative result, and you are speculating that there is a novel transport mechanism that has not really been demonstrated.

We agree with this reviewer that this was overly speculative. We have removed the sentence.

Referee #2 Major issues:

As the paper is written, it is showing novelty and biological significance, but is weak showing behavioural evidence to the pain field. In other words, the paper clearly demonstrates that GIRK channels are absent in wild type mice (structural biology, in vitro functional electrophysiology), but the way pain research methods were used are not good enough to demonstrate robust in vivo confirmation (in vivo functional biology); making hard to see that the nonexistence of these channels fully accounts for the difference between opioid-mediated analgesia in mice versus opioid-mediated analgesia in rats or humans.

In addition to other experiments, we have now added a large quantity of behavioural data to the manuscript as detailed below.

The behavioural findings are supported only by few experiments and incomplete description of the nociceptive techniques implemented. Behavioural studies compared baseline responses against two days after CFA injections for each mice group only (Fig. 4). It is well known that after a day of a

single CFA injection the observed hyperalgesia response decays progressively. It would be better to repeat the assay and test the animals two hours after the CFA injection when they must show the greatest hyperalgesia seen in this model, or to substitute the CFA test with the formalin test.

We have re-analysed a substantial amount of behavioural data from our laboratory demonstrating that hyperalgesia does not significantly differ between 2 hours and 2 days after CFA injection (Figure for reviewers). In addition, we do not believe that the formalin test is a suitable substitute for CFA inflammation. This is because ultra-short acting stimuli such as formalin (less than an hour) do not appropriately mirror clinical pain, which is typically associated with manifest tissue inflammation and lasts for at least several days. Many publications have extensively reviewed and discussed the predictive value of animal models for the clinical situation and have come to similar conclusions (e.g. Stein et al. Pharmacol Biochem Behav 1988;31:445-451; LeBars et al. Pharmacol. Rev. 2001;53:597-652; Mogil. Nat. Rev. Neurosci. 2009;10:283-294; Berge. Br. J. Pharmacol. 2011;164:1195-1206).

Authors used the CCI method to induce neuropathy in mice, but they do not show any behavioural data describing their results, only a follow up of two days checking for the GIRK2 mRNA expression in DRG neurons (Fig. S2). The full development of the mechanical and thermal hyperalgesia seen in the CCI model takes more than two days for both rats and mice. I suggest changing the CCI for SNI (spared nerve injury) model which has a better output in mice and to let the neuropathy to develop for at least 10 days.

We have added a substantial amount of new data demonstrating that both mechanical and thermal hyperalgesia are indeed fully developed at 2 days and do not significantly differ from 14 days after CCI (Figure for reviewers). We have used the CCI model because it is one of the oldest and most thoroughly characterized animal model for neuropathic pain (Jaggi et al. Fundamental & Clinical Pharmacology 2011;25:1–28), and because it has been used in many publications examining peripheral opioid analgesic effects (reviewed in Stein & Machelska, 2011). In terms of predictive value and comparability, we and others believe that the CCI model is closer to the clinical situation than the SNI model. This is mainly because in humans partial nerve injury (such as CCI) is a more common scenario (e.g. carpal tunnel syndrome, tumour compression, spinal disk prolapse, nerve entrapment, extremity trauma) than a complete nerve transsection (such as SNI) (e.g. amputation). Several reviews have come to similar conclusions (Kontinen and Meert. Proc. 10th World Congress on Pain. IASP Press, Seattle 2003;489-498; Jaggi et al. Fundamental & Clinical Pharmacology 2011;25:1–28). In addition, the SNI model was originally developed to enable a direct way to study changes in both injured and neighbouring intact sensory neurons (Decosterd and Woolf. Pain 2000;87:149-158). In the original investigation, sensory testing was differentially performed on medial versus lateral, and dorsal versus ventral parts of the rat paw (sural, common peroneal and saphenous nerve territories). This was not the aim of our study and would likely complicate the interpretation of our data, particularly since we are using mice with much smaller paws. In addition, we would have to apply for approval of this completely new model by our ethical committee, which would require a minimum of 4-6 months. In summary, we feel that the expected minimal gain in knowledge would not justify the costly establishment of this new model in our laboratory.

It would be a great value to the paper to divide and to describe behaviour results in two groups: acute inflammatory pain and chronic neuropathic pain and once hyperalgesia is confirmed then proceed to test opioid mediated analgesia in both; also to add behaviour data done in rats as an important standard point of comparison.

We have now added a substantial amount of new data demonstrating the effects of DAMGO and NLXM in rats with CFA inflammation. We do not have an approved animal protocol for CCI in rats. To get a new protocol approved by the ethics committee would take at least 4-6 months. However, the reviewer's point is well taken and we are now planning such experiments within a separate new project. As such though, the focus of the current paper is CFA inflammation.

<u>Minor issues:</u>

Subjects: mice strains used in this study are described in the results and not in the methods. Neither rat strain(s) used is/are described nor the origin of human samples used (e.g. where they came from, if the donors were healthy or not, etcetera).

We have added this information to the text

Pain assessment: this subsection in the methods must be rewritten, as it is, is not solid. It would be better to describe first, all species used (sex, ages...); second, basal nociception tests, inflammatory

test, neuropathic test; and third, the intraplantar pharmacology. Extra details in this subsection will be beneficial as the paper title lays on opioid-mediated analgesia with is directly linked to (in vivo) behavioural pharmacology.

The methods section has been rewritten.

Referee #3 (General Remarks):

Specific points:

1) Fig1G: too dim signal to have an opinion. Please provide convincing example.

We have added a new figure to 1G.

2) The reference to a "functional" expression of GIRK2 channels in human (in the text and in the figure2) is not supported at all. The only evidence provided is an example of labeling in fig2g. This is largely insufficient to claim that this is a functional expression in human DRGs. The author should be more convincing by showing more staining using fluorescence immunodetection as for mice sections. The expression in human DRG would be required also to claim that the channel is expressed in DRG. In addition the analysis done on the rat and mouse promoter region of GIRK2 should be also performed in human to conclude in the text that rat and human have a conserved mechanism.

We agree with the reviewer's comment and have therefore initiated a series of experiments examining the functional expression of GIRK2 channels in human DRG neurons in more detail. Unfortunately, it is not easy to obtain sufficient amounts of human DRG neurons. We have established a protocol with The National Disease Research Interchange in December 2012 and have received 6 human DRGs just last week. Thus, we anticipate at least another 4-6 months before we can complete these experiments. At this point we have therefore decided to remove the passages related to human DRG neurons and to tone down our respective conclusions. Upon completion of our ongoing experiments we plan to draft a separate manuscript.

We expanded our analysis of GIRK promoters to the human. We attempted to express human GIRK2 promoter reporter constructs in mouse DRG but were unable to detect fluorescence, most likely because this region of DNA is not well conserved between humans and rodent, making it difficult to align homologous regions. With this in mind we also performed an analysis on the GIRK1 promoter which is more similar between humans and mouse. However, using up to 2kb of sequence prior to the transcription site of GIRK1 we were unable to drive expression with either mouse, rat or human constructs. We thus conclude that the short regulatory sequence we observed in the rat GIRK2 promoter is sufficient to explain differences in expression between rodent species, but not to humans. This has been added to the text.

3) The expression of the GIRK2-flag channels in the transgenic animals should be better described. It is hard to identify the neuronal DRG subtypes targeted by the transgene. A classical fluorescent immunodetection of the flag epitope together with nociceptive and non-nociceptive markers in DRG sections would be more appropriate (Flag + IB4, + CGRP, +NaV1.8, +peripherin, +NF200, etc...).

We have performed additional fluorescent immunohistochemistry using flag antibody in conjunction with IB4, CGRP and NF200 staining. This is now included in Figure 3.

4) Since the transgene is not expressed in all DRG neurons, the authors should explain how they identify the neurons they are patching.

Neurons were patched blindly. All cells were included in the analysis.

5) The authors provide evidence for a preferential peripheral expression of GIRK2 in the transgenic animals with a minimal if any targeting to the central synapse in the sensory neurons. Again the IHC data provided with DAB staining of the flag epitope are the only arguments. Other arguments should be provided such as dual immunodetection of the flag and the GIRK2 (anti flag and anti GIRK2 with 2 different fluorescent secondary). A western blot with an anti-flag on skin / sciatic nerve / DRG / and spinal cord should be presented. Ultimately, the use of the AAV-GIRK2 in vivo (injection in the sciatic nerve), would allow to see if the GIRK2 protein is restricted to the

peripheral part of the DRGs or not. Such absence of functional GIRK2 from the presynaptic sensory inputs in the spinal cord could be also probed by electrophysiological means in slices by comparing the DAMGO modulation of mini EPSP in WT and Tg animals.

We were initially overly speculative in our assertion that GIRK2 is preferentially trafficked to peripheral terminals of sensory neurons. As made clear by these reviews, multiple lines of evidence are needed to substantiate this claim, such as a thorough histological examination, peripheral overexpression and ultimately electrophysiological analysis of spinal cord slices. This amounts to a separate study in itself and detracts from the main aim of the current work. Accordingly we have removed this section from the text.

6) The behavioral data shows that the analgesic doses of DAMGO are totally different in thermal and mechanical pain stimuli paradigms. This is rather spectacular and not discussed. An explanation should be provided. What kind of effect is achieved with 48µg on mechanical pain?

We have now discussed this in depth in the manuscript.

7) The method for transfecting DRG neurons in culture with the promoter-GFP plasmids is not explained.

We used the Amaxa Nucleofector II System with the Basic Neuron SCN Nucleofector Kit following the manufacturer's protocol. This is indicated in the methods

<u>Minor point:</u>

1)Sup figure 1: please show tail current on the calcium channel traces as well as full time course of the experiment with DAMGO washout

This has been added.

2)Provide reference for the well-known expression of GIRK2 in keratinocytes. Staining in Girk2 KO?

This sentence was inaccurate and has been changed.

Figure for reviewers:

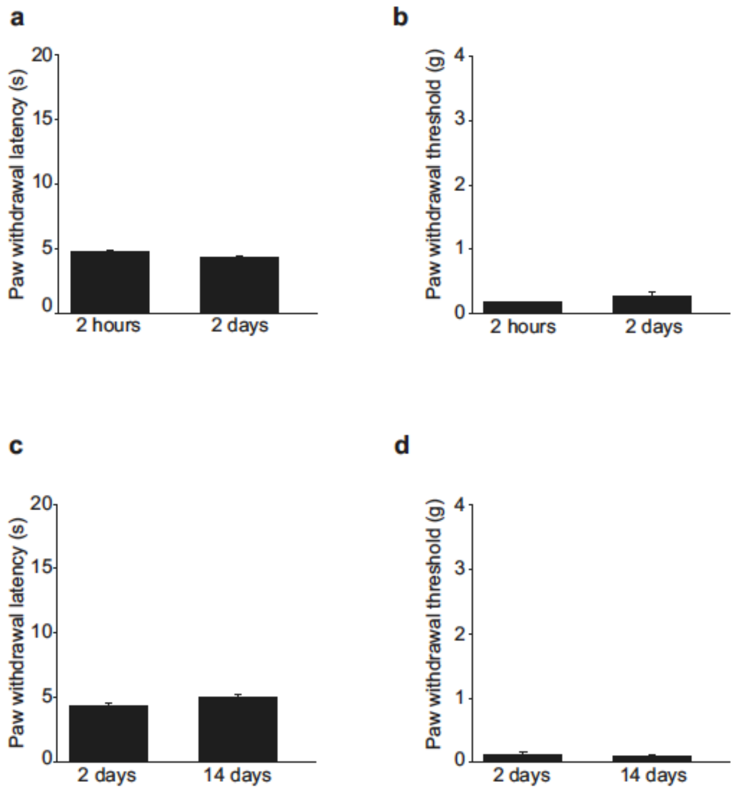

2nd Editorial Decision 16 May 2013

Thank you for the submission of your revised manuscript to EMBO Molecular Medicine. We have now received the enclosed reports from the Reviewers that were asked to re-assess it. As you will see the reviewers are now supportive and I am pleased to inform you that we will be able to formally accept your manuscript pending the following final amendments:

1) As per our Author Guidelines, the description of all reported data that includes statistical testing must state the name of the statistical test used to generate error bars and P values, the number (n) of independent experiments underlying each data point (not replicate measures of one sample), and the actual P value for each test (not merely 'significant' or 'P < 0.05').

2) I would like to take out "in mice" from the title. Would this be accptable? Otherwise you can propose an alternative as long as "mice" is not in the title and, of course, the title is appropriate!

Please submit your revised manuscript within two weeks. Needless to say, the sooner we receive it the sooner I will be able to formally accept your manuscript.

I look forward to reading a new revised version of your manuscript as soon as possible.

***** Reviewer's comments *****

Referee #1 (General Remarks):

The authors have nicely improved the paper in response to several of my prior comments (and have gently explained why other comments were off base). The paper's basic discovery is exceptionally well documented: G-protein coupled potassium channels are the molecular effector for opioid-induced K currents in rat sensory neurons. The work also shows that this mechanism largely explains why opioids cause peripheral analgesia in rats but not mice. I recommend publication in its present form.

Referee #2 (Comments on Novelty/Model System):

Most of my previous suggestions and pertinent corrections about pain behavioral experiments and pain animal models have been added to the manuscript and those which not have been refuted appropriated. Therefore, I consider the paper suitable for publication if the Authors have addressed others peer-evaluations accordingly and my colleagues are satisfied too.

Referee #3 (Comments on Novelty/Model System):

The revised version of the manuscript is cleally improved and the suggestions made on the initial version has been taken into account.
I do not have any more objections to this work that is suitable for publication to my opinion.

Referee #3 (General Remarks):

The revised version icorporated the suggestions made on the initial review. The manuscript is clearlly written and the discussion fair and of interest for the readership of EMBO molecular medicine. Although I would have like to see more immunological data on human samples, I understand that this will be a study on its own in the future. I think that the manuscript is suitable for publication as it is now.

2nd Revision - authors' response 24 May 2013

Please find attached a resubmission of our manuscript entitled "The K+ channel GIRK2 is both necessary and sufficient for peripheral opioid-mediated analgesia".

We have amended the manuscript as requested.
